# Structural, electronic and magnetic properties of the ordered binary FePt, MnPt, and CrPt_3_ alloys

**DOI:** 10.1016/j.heliyon.2020.e03545

**Published:** 2020-03-06

**Authors:** A. Alsaad, A.A. Ahmad, Tareq S. Obeidat

**Affiliations:** Department of Physics, Jordan University of Science and Technology, P. O. Box 3030, Irbid 22110, Jordan

**Keywords:** Materials science, Condensed matter physics, Ordered binary alloys, Magneto-crystalline anisotropy energy (MAE), Spin density functional theory, Magneto-crystalline anisotropy constant, Force theorem, Structural properties, Electronic properties

## Abstract

We perform ab initio simulations to investigate the structural, electronic and magnetic properties of the ordered binary FePt, MnPt, and CrPt3 alloys. In particular, equilibrium structural lattice parameters, electronic properties such as density of states (DOS), partial density of states (PDOS) and electronic band structure of each binary alloys are investigated and interpreted. Moreover, the magneto-crystalline anisotropy energies (MAE) are calculated. We found MAE values of FePt, MnPt and CrPt_3_ ordered alloys to be 2.66, 0.46 and 0.42 meV/f.u., respectively, corresponding to magneto-crystalline anisotropy constant K of 7.6 × 10^7^, 1.3 × 10^7^ and 1.1 × 10^7^ erg/cm^3^, respectively. The large MAE and K values reveal that FePt, MnPt and CrPt_3_ binary alloys are eligible to be key components in magneto-optical and opto-electronic devices. In addition, we estimated the Curie temperatures of the three ordered alloys from exchange energy. We found the T_C_ of L1_0_-FePt, L1_0_-MnPt and L1_2_ CrPt_3_ to be 955 K, 989 K and 762 K, respectively. The high Curie temperatures obtained enable the ordered alloys to serve as write assist in Heat-Assisted Magnetic Recording (HAMR). We believe that our findings would pave the way to fabricate bulk and thin films based on the ordered binary FePt, MnPt, and CrPt_3_ ordered alloys that have attractive electronic and magnetic properties.

## 1 Introduction

In the past two decades, the interest in hybrid materials formed by coupling transition metals with platinum (Pt) has attracted much attention both experimentally and theoretically due to their excellent structural and magnetic properties. In particular, high chemical and thermal stabilities, good display performance and a large magneto-crystalline anisotropy (MAE) of the ordered binary FePt MnPt, and CrPt_3_ alloys made them superior to other binary alloys used in magneto-optical recording devices [1, 2, 3, 4] communication devices [5], optical filters [6], biomedical applications [7] and spintronic applications [8, 9]. The FePt and MnPt binary alloys are considered as potential candidates for state-of-art spintronic applications like magnetic random access memory (MRAM) [10, 11] and drivers in micro- and nano-electromechanical systems (MEMS/NEMS) [12,13,14,15,16]. It has been reported that high-purity L1_0_ FePt NPs could be obtained by controlling the proportion of iron and platinum only in the vicinity of the equiatomic composition [17]. Several works have focused on the investigation of FePt thin films and related materials [18, 19, 20, 21]. First-principles based calculations addressing the magnetic and electronic properties of L1_0_ FePt and MnPt alloys have presented interesting results [22]. The out-of-plane ground state Magnetocrystalline anisotropy energy (MAE) of MnPt was found to be more than an order of magnitude (~0.1 meV/f.u.) less than that of FePt (~2.9 meV/f.u.) [23, 24]. The synthesis and characterization of a well-organized magnetic selection of such materials can promote the design of magnetic media sufficiently proficient of recording densities beyond 1 Tb/in^2^ [25,26].

**Figure 1:**
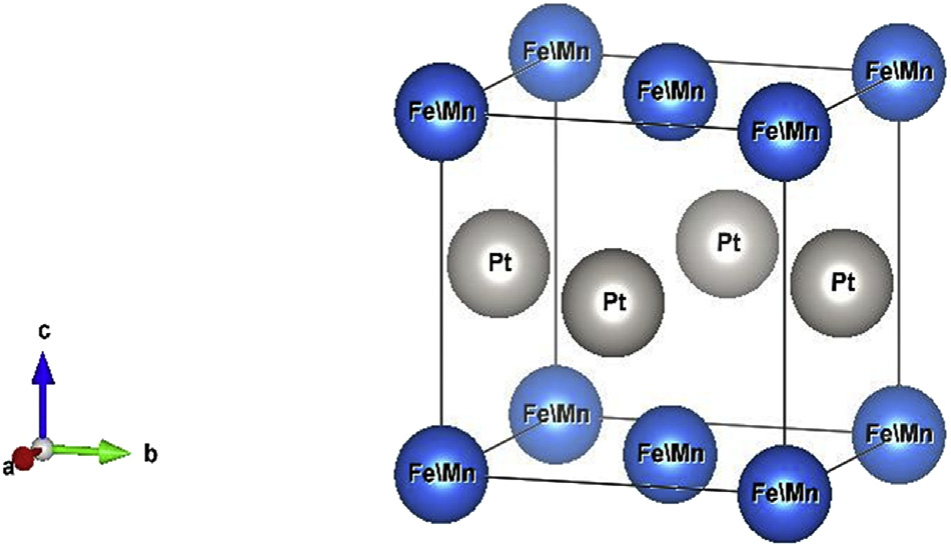
Schematic diagram of fct AuCu (L1_0_) phase of FePt and MnPt ordered binary alloys.

**Figure 2:**
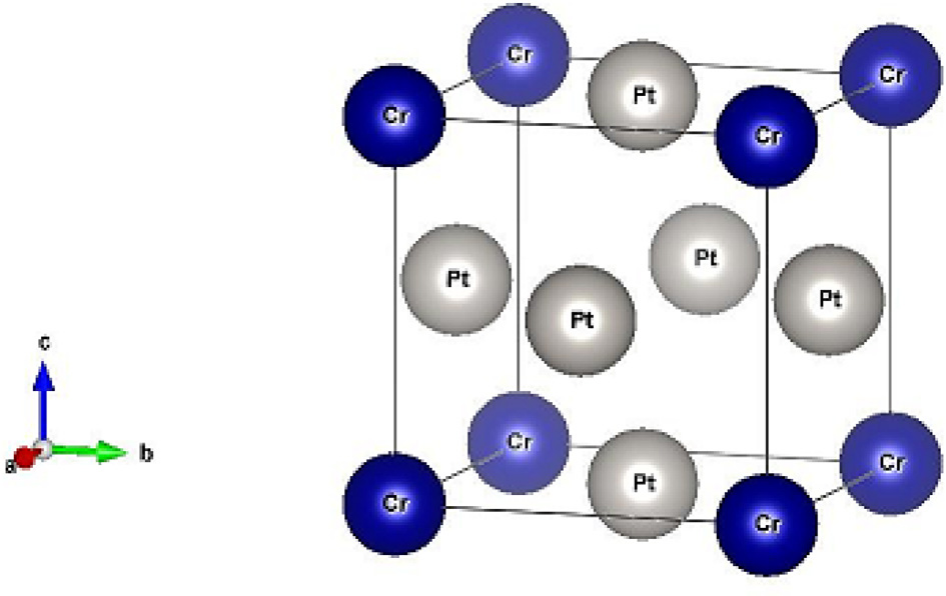
Schematic diagram of AuCu_3_ (L1_2_) structure of CrPt_3_ ordered alloys.

The MAE is defined as the change in the total energy associated with a change of the orientation of the spin moment along different crystallographic axes of the crystal. The contributions to MAE are two-fold: The first is the volume shape anisotropy and the second is magnetocrystalline anisotropy. The shape anisotropy is a long-range effect depends on the shape of the crystal arise from the dipole-dipole interactions that favor always the in-plane orientation of the magnetization axis [17]. The MAE arises mainly from the spin-orbit interaction which is a short-range effect localized around atomic cores [18,20,21]. Moreover, MAE indicates the degree of magnetic stability and is related to the value of the spin magnetic moment of certain ferromagnetic materials [19]. In this work, we investigate the structural, electronic and magnetic properties of the ordered binary FePt, MnPt, and CrPt_3_ alloys that crystallize in L1_0_ (also known as AuCu) and L1_2_ (known as AuCu_3_) as illustrated schematically in Figures 1 and 2.

It is well known that the value of MAE can be tuned over broad range by varying *c/a* ratio [24]. Experimental investigations have reported *c/a* ratio to be 0.96, 0.92 and 1.00 for FePt, MnPt, and CrPt_3_ alloys, respectively [25, 26, 27]. Several theoretical calculations and experimental measurements have been performed to elucidate the values of MAE of L1_0_-like Fe_x_Pt_1−x_ structures. *C.J. Aas* [28] performed first-principles calculations of the MAE of the L1_0_-like Fe_x_Pt_1−x_ samples. Several theoretical studies have used the coherent potential approximation (CPA) [18, 29, 30] to determine the MAE of partially ordered alloys. Furthermore, a pioneering experimental study by Barmak et. al [31] reported an epitaxial growth of FePt films with nominal thicknesses of 42 or 50 nm by sputtering onto single crystal MgO(001) substrate. The calculated MAE was found to be in the range 0.453–0.775 meV/f.u. at Fe concentration 46%–66%. Rie Y. Umetsu [32] presented theoretical calculations of MAE of the L1_0_-type MnPt alloy using local spin-density approximation (LSDA) and implementing the Linear Muffin-Tin Orbital (LMTO) method with atomic sphere approximation including the spin-orbit interaction [3]. The magnitude of the MAE of L1_0_-type was estimated to be about 0.51 meV/f.u. This corresponds to a magnetocrystalline anisotropy constant K of approximately 1.39 × 10^7^ erg/cm^3^. A pioneering theoretical study reveals that the CrPt_3_ is the most stable phase of the Cr_x_Pt_1-x_ system and it exhibits an abnormally large MAE [2, 25, 33]. This inconsistent with the fact that the most stable phase of CrPt_3_ is the cubic AuCu_3_ structure, in which symmetrical considerations would discard a large MAE value [34, 35, 36]. The large MAE value of CrPt_3_ systems has attracted a great deal of interest since such materials can be used in the fabrication of novel magneto-optical devices. P.M. Oppeneer [2] examines computationally the origin of the unique magnetism observed in crystalline CrPt_3_ films and found that CrPt_3_ has a large orbital moment of 0.15 μ_B_ develops on Cr parallel to the spin moment of 2.72 μ_B_. The MAE in the cubic 3d transition metals is a very small quantity of only a few μeV/f.u. [36, 37, 38]. In light of the above, we are motivated to calculate MAE of the three ordered alloys with high degree of accuracy and to interpret the physical origin of such large values.

## 2 Computation details

We have done self-consistent electronic structure calculations that are predominantly carried out within the spin-polarized DFT approach implemented in the Vienna *ab initio* simulation package (VASP) [37, 38, 39, 40]. The Projector Augmented Wave (PAW) pseudopotentials [41, 42, 43] are used to describe electron-ion interactions. The electronic configuration of the metals involved in the three ordered binary alloys are as follow: Fe: 3d^7^ 4s^1^, Mn: 3d^6^ 4s^2^ Cr: 3d^5^ 4s^1^ and Pt: 5d^9^ 6s^1^, respectively. The exchange-correlation functional [44] is accounted for by using the spin-polarized generalized gradient approximation (GGA) as parameterized by Perdew–Burke–Ernzerhof (PBE) [45, 46, 47].

Previous studies have emphasized the importance of k-sampling in the full Brillouin zone to get stable and reliable results of MAE of FePt alloys [48]. Consequently, we used large supercell to ensure high accuracy in determination of MAE values. We did carry out sets of calculations of all binary alloys with a mesh-grid of 24×24×24 k-point corresponds to a slightly less than 14.000 k-points. We use an energy cuto-ff of 300 eV. The spin-orbit coupling was taken into account only for the calculation of MAE using the force theorem [49, 50]. MAE is calculated in terms of the difference in band energies of the two magnetization directions with spin-orbit coupling included in the Kohn-Sham equation. Two sets of self-consistent calculations were performed, one with magnetic moments aligned parallel “$E_{∥}$”and the other oriented perpendicular” $E_{⊥}$ “to the easy axis (*c*-axis). According to this approach:

$$MAE=E_{∥}-\;E_{⊥}.$$

We employed a model based on simultaneous use of force theorem and LSDA to compute the MAE of all the binary structures investigated in this work. The justification of implementing this approach is vindicated by the vanishing charge- and spin-density variations confirming that the spin-orbit coupling is correctly computed [44]. The LSDA method is very simple and promising for first principles determinations of MAE. In this method, on-site Coulomb potential depends on spin only. More precisely, LSDA is the best for transition-metal compounds where the spin-orbit coupling generally lowers the symmetry of a magnetic system compared to its crystalline symmetry. The local spin density + Hubbard U approximation (LSDA + U) was shown to provide a reliable representation of the magnetic ground state features when Coulomb correlations among atomic species are included for both 3d and 5d elements in FePt [51]. The electron-electron interaction plays a crucial role in the determination of MAE in *d*- [52] and *f*-electron [53] magnetic materials. The pioneering study implemented the LSDA + U method and full potential linearized augmented plane wave method [53, 54] to describe the Coulomb repulsion U among different atomic species. The main idea of LSDA + U method is that “+U” potential has both onsite spin- and orbital-contributions. The charge/spin densities are converged better than 5 × 10^−5^ electron/(a.u.)^3^ in order to accomplish accurate total energy convergence. In calculating the ionic relaxation (relaxed geometry), we performed a self-consistent (SC) calculation using a conjugate gradient algorithm (CG) [55, 56, 57]. We constructed the unit cell of each of the investigated alloys by starting up with experimental lattice parameters (*a* = 3.86, *c/a* = 0.96, *a* = 4.00, *c/a* = 0.92 and *a* = 3.87, *c/a* = 1) for FePt, MnPt and CrPt_3_ alloys, respectively [3, 25, 27]. All the structures are then fully relaxed as described above. For FePt and MnPt, a 9 × 9 × 9 Monkhorst–Pack k-point mesh [58, 59] and an energy-cutoff of 300 eV were sufficient for energy convergence while for the CrPt3, we used a 6 × 6 × 6 Monkhorst–Pack k-point mesh. The Methfessel Paxton method [60, 61] is implemented for all structures to speed up the integration over the Brillouin zone. The relaxed atomic positions were followed by minimizing the total energy (i.e., as small as 10^−6^ eV), and the Hellmann–Feynman forces. These forces were as small as 0.0003 eV/Å at convergence in the unit cell of all different electronic structure relaxations that has been performed. Furthermore, to ensure high accuracy of calculating the DOS of each alloy, we used 18 × 18 × 18 Г–centered Monkhorst Pack k-point mesh with an energy cut-off of 400 eV.

## 3 Results

### 3.1 Crystal structures and total energy minimization approach

**Figure 3:**
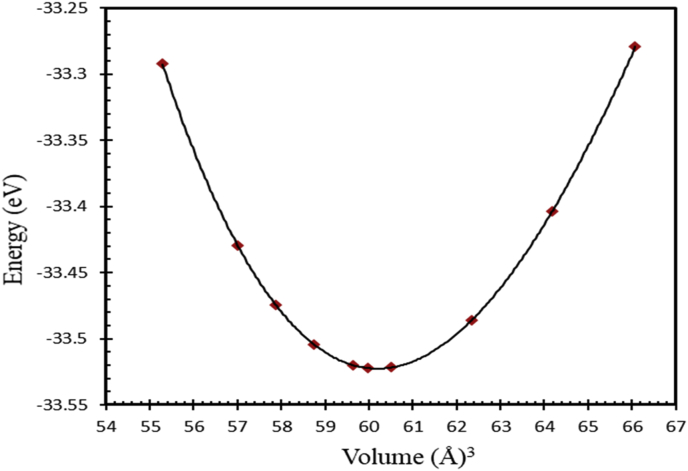
Total energy of the unit cell versus volume of FePt binary. Birch-Murganan (B–M) equation of state has been used to fit the data.

**Figure 4:**
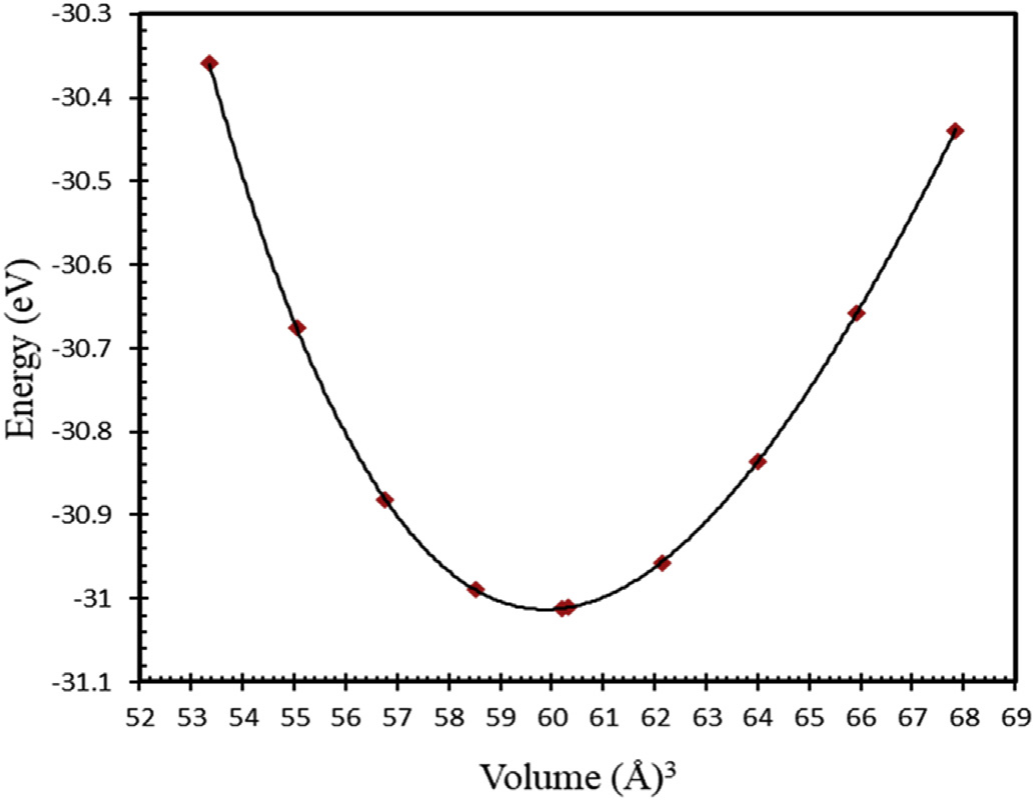
Total energy of the unit cell versus volume of MnPt binary. Birch-Murganan (B–M) equation of state has been used to fit the data.

**Figure 5:**
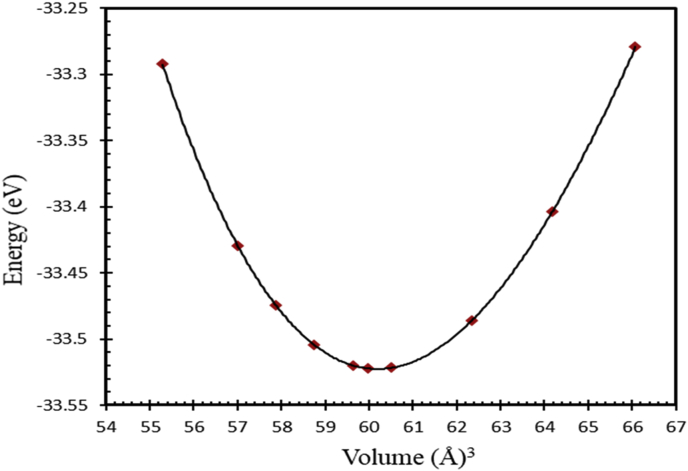
Total energy of the unit cell versus volume of CrPt_3_ binary. The Birch-Murganan (B–M) equation of state has been used to fit the data.

Generally, L1_0_ MPt [M = Mn, Fe] structure consists of two types of atoms as shown in Figure 1. Mainly, Mn or Fe atoms occupy the [0 0 0] and [½ ½ 0] lattice sites and Pt atoms occupy the corresponding face center with [½ 0 ½], and [0 ½ ½] site positions [24, 62, 63], respectively. As shown in Figure 1, both FePt and MnPt exhibit a distorted tetragonal structure labeled by L1_0_ with space group (P4/mmm). Total energy minimization plots of L1_0_ phase of FePt and MnPt ordered alloys are shown in Figures 3 and 4. The obtained lattice parameters were found to be *a* = 3.88 Å, *c* = 3.73 Å, *c/a* = 0.961 and volume = 56.1 Å^3^ for FePt ordered alloy and, *a* = 4.03 Å, *c* = 3.69 Å, *c/a* = 0.916 and volume = 60 Å^3^ for MnPt ordered alloy, respectively. As illustrated in Figure 2, CrPt_3_ was found to exhibit the cubic L1_2_ phase with space group (Pm3m). In L1_2_ structure Cr and Pt atoms are located at the corner and face center, respectively 2 with [0 0 0] for Cr and [½0½], [½ ½ 0], and [0 ½ ½] lattice sites for Pt [64, 65]. Total energy minimization plot for L1_2_ structure is shown in Figure 5. The calculated lattice parameters of the L1_2_ structure was found to be, *a* = *c* = 3.91 Å and volume = 60.2 Å^3^.

The Antiferromagnetic (AFM) of MnPt and Ferromagnetic (FM) FePt phases have been intensively studied in past two decades due to their inherited outstanding properties. They were used for quantum computing applications and high-density magnetic recording. Both MnPt and FePt binaries crystallize in teterahederal distorted L1_0_ (CuAu) atomic structure. Total energy minimization approach is used to calculate the equilibrium lattice constants of both binaries, as well as, (CuAu_3_) CrPt_3_ ordered alloys. The calculated equilibrium lattice constants of the three ordered binary alloys are listed in Table 1.

**Table 1:** Equilibrium lattice constants of L1_0_ (CuAu) FePt and MnPt, as well as and L1_2_ (CuAu_3_) CrPt_3_ ordered alloys.

Material	a(Å)	c(Å)	a_ex_ (Å)	c_ex_(Å)	a_th_(Å)	c_th_(Å)	c/a	c/a_ex_	c/a_th_
FePt	3.88	3.73	3.86^a^	3.71^a^	3.87^b^	3.73^b^	0.96	0.96^a^	0.96^b^
MnPt	4.03	3.69	4.00^c^	3.67^c^	3.99^d^	3.70^d^	0.92	0.92^c^	0.93^d^
CrPt_3_	3.91	3.91	3.87^e^	3.87^e^	3.92^f^	3.92^f^	1	1^e^	1^f^

a Ref. [27].

b Ref. [62].

c Ref. [3].

d Ref. [24].

e Ref. [25].

f Ref. [63].

The details of the crystal structures and total energy minimization approach implemented for obtaining the optimized lattice parameters of the three ordered magnetic alloys are provided in the supplementary of this manuscript.

### 3.2 Density of state and band structure (BS) of FePt, MnPt, and CrPt_3_ binaries

**Figure 6:**
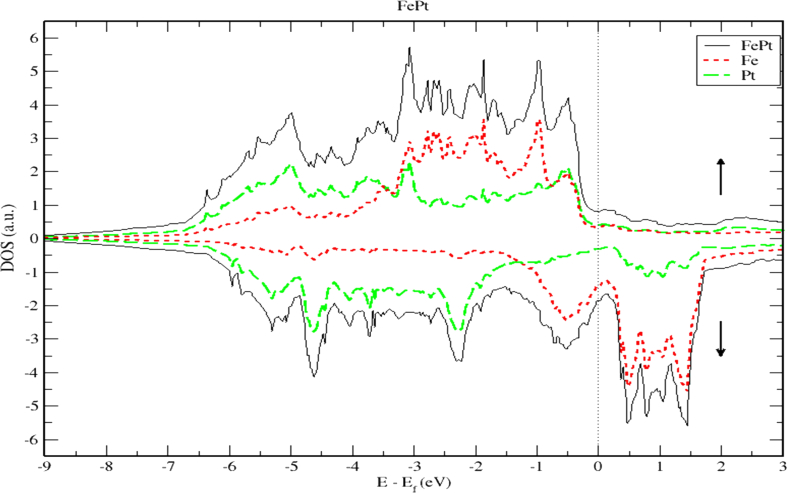
Density of state of FePt binary. The dashed lines represent partial density of states (PDOS) of Fe and Pt elements. The vertical line corresponds to the Fermi energy.

**Figure 7:**
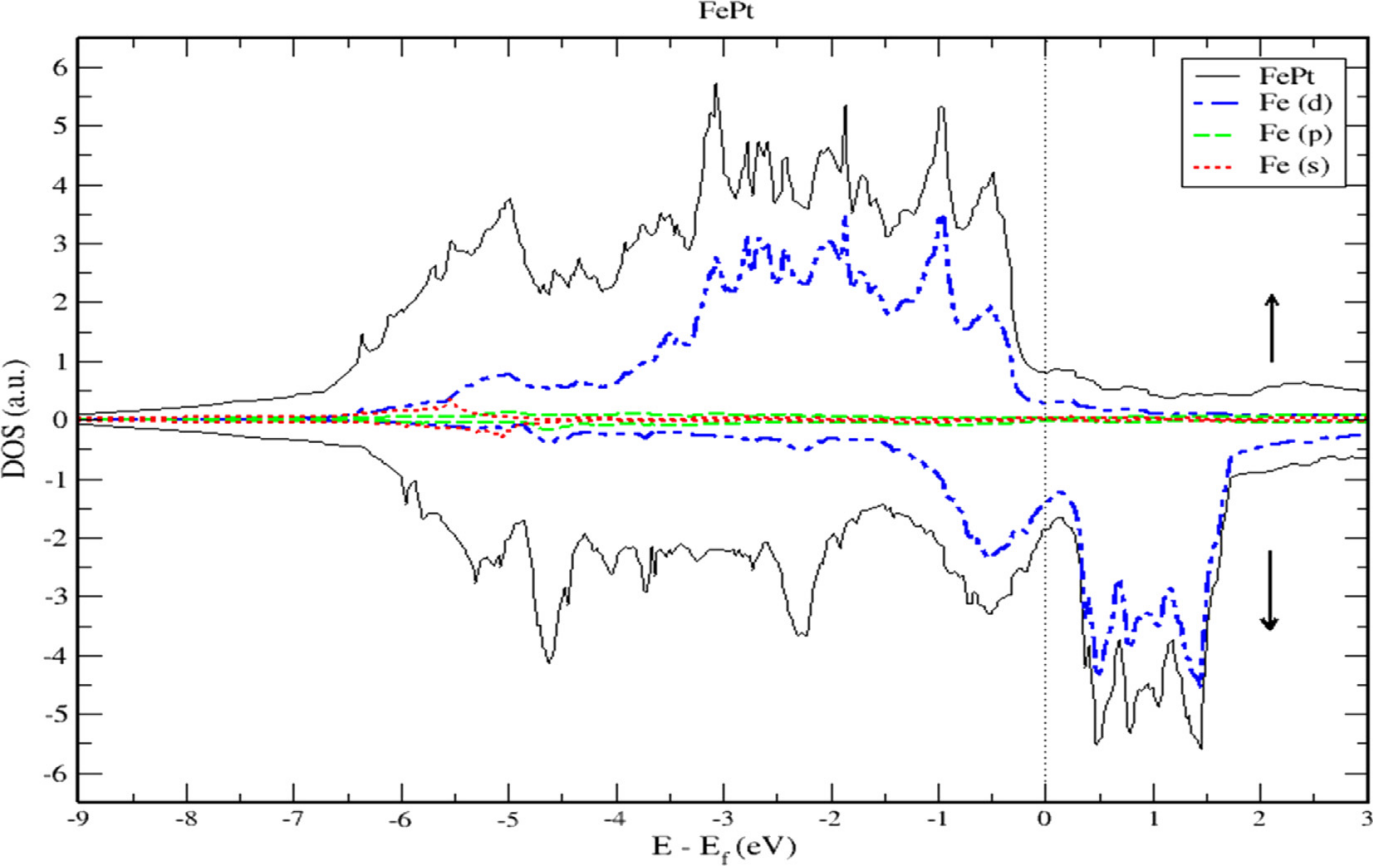
Density of state of FePt binary. The dashed lines represent partial density of states (PDOS) of Fe. The vertical line corresponds to the Fermi energy.

**Figure 8:**
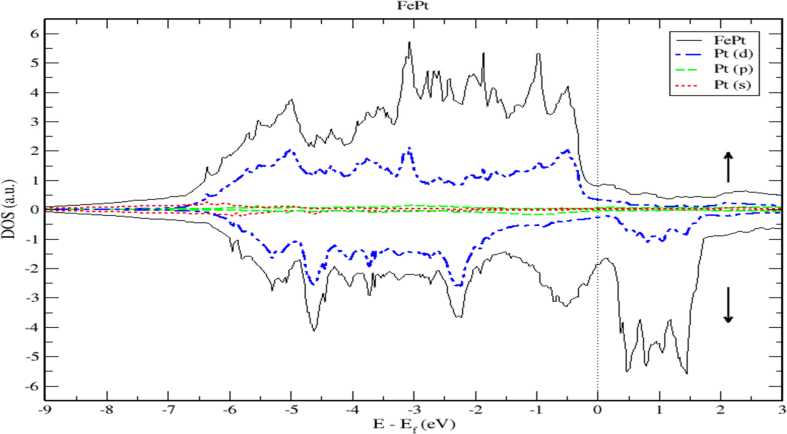
Density of state of FePt binary. The dashed lines represent partial density of states (PDOS) of Pt. The vertical line corresponds to the Fermi energy.

**Figure 9:**
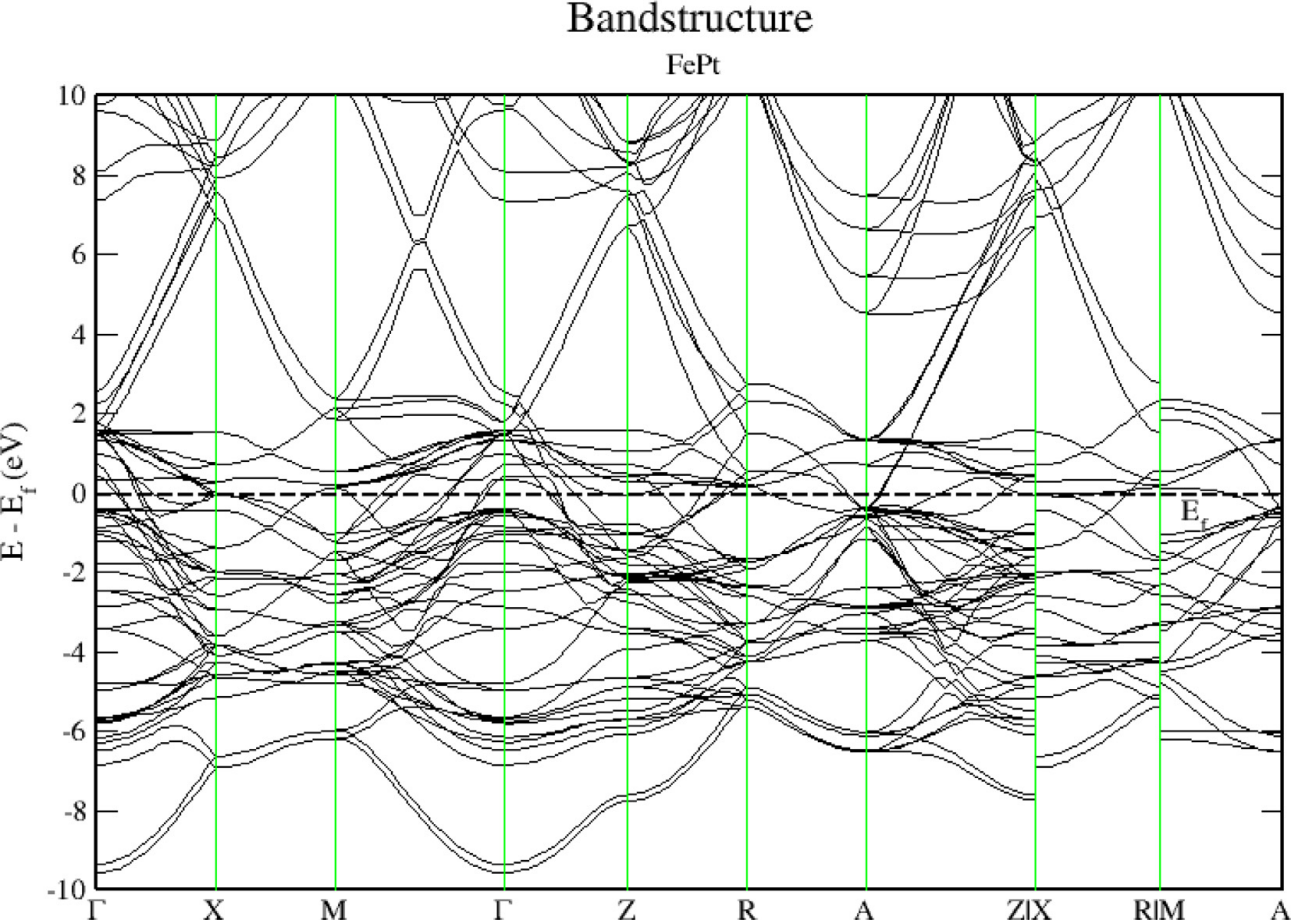
Electronic band structure of the FePt binary. Band gap is missing, and bandgap energy is identically zero.

**Figure 10:**
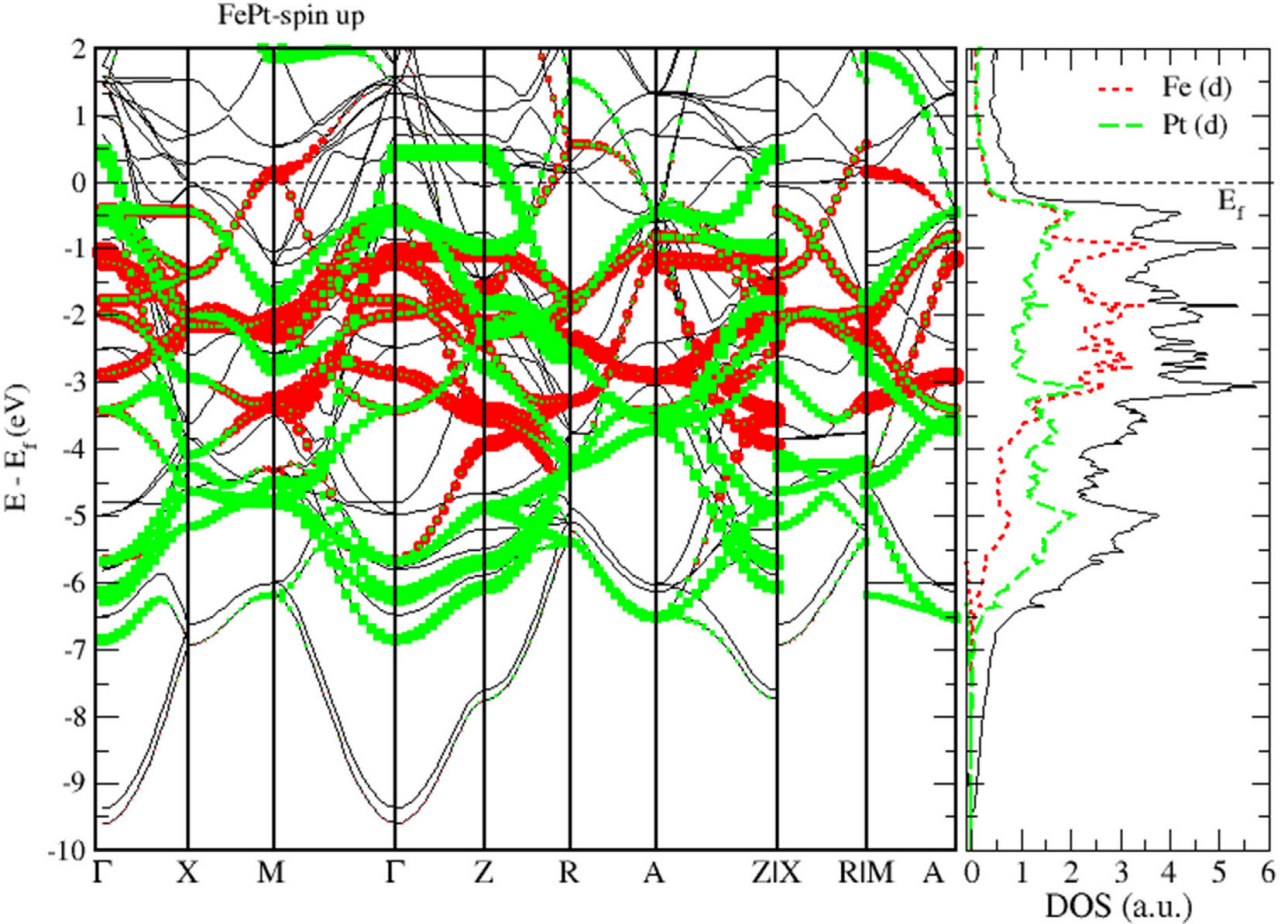
Band structure and spin up PDOS of FePt binary.

**Figure 11:**
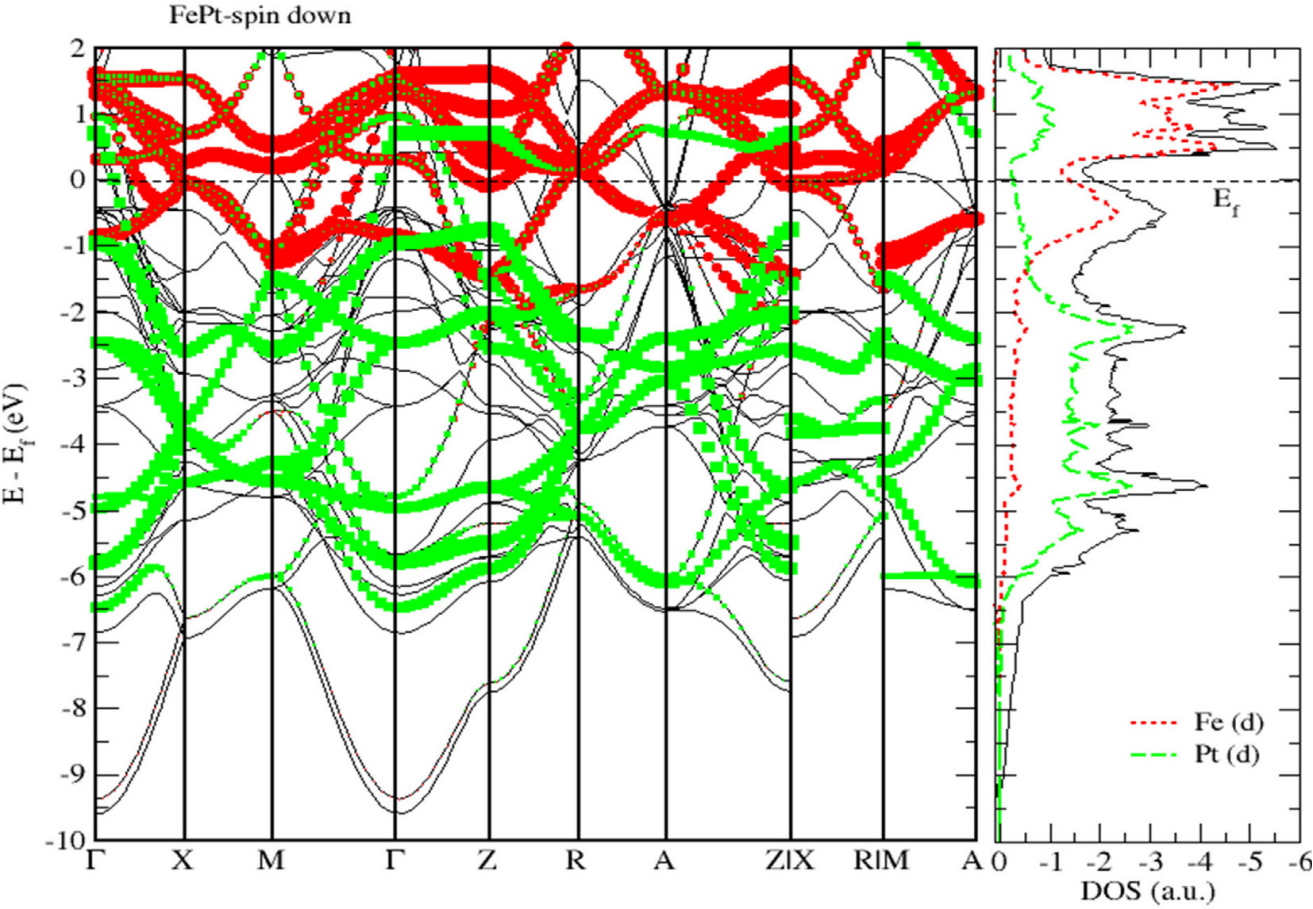
Band structure and spin down PDOS of FePt binary.

To obtain a deeper insight into the electronic properties of FePt, MnPt, and CrPt_3_ ordered alloys, an accurate investigation of electronic DOS and BS are important. Figures 6, 7, 8, 9, 10, and 11 show our results of the total density of state (TDOS), partial density of state (PDOS) and band structure of the FePt compound. Figure 9 shows the TDOS where the Fermi level is set to 0 eV. The contributions to TDOS of FePt come mainly from the hybridization of Fe-3d and Pt-5d electronic states while the s and p electronic states contributions are found to be negligibly small as shown in Figures 7 and 8. As indicated by figures, the highest DOS of the majority spin "spin up" valence bands, are characterized by energy values located at ~ -3.1 eV, ~ -1.9 eV, and ~ -0.9 eV whereas, the highest DOS of the minority spin “spin down." conduction bands, are found at ~ 0.46 eV, ~ 0.78 eV, and ~1.44 eV. We found that the width of the majority band ~6 eV is narrower than the corresponding energy width of the minority band ~ 8eV which means that the DOS is larger in the majority bands than in the minority bands. The behavior of TDOS near E_f_ depends largely on the contribution of Fe-3d states and exhibits a low value near the Fermi energy where the lower energy part of the Fe-3d states are almost empty and the Pt-3d states are almost completely occupied. Therefore, the contribution of one-spin channel of the Fe-3d states electrons to the hybridization with electrons of neighboring atoms is extremely small. This weaker hybridization leads usually to the narrowing of the bandwidths and enhancing the exchange splitting. Furthermore, we present the projected DOS for the Fe, Pt atoms and FePt bulk as shown in Figures 6, 7, and 8. The d bands of both Fe and Pt significantly overlap below E_f_ in the majority spin state indicating that both d bands in the majority spin state strongly hybridize with each other. Therefore, ferromagnetic spin configuration of FePt originates from the d-d hybridization between Fe and Pt. However, the minority spin state, d bands of Pt and Fe form bonding and anti-bonding states respectively; this is where hybridization comes into place.

Figure 9 displays the electronic band structure of FePt system. It is obvious that the FePt system is metallic in nature. By careful inspection of Figures 10 and 11, we can distinguish three regions of interest. Mainly, the region dominated by Pt-5d electronic states in the (-7 to -4 eV) range below E_F_ level. The second region characterized by the strong hybridization of Pt-5d states and Fe-3d states in the energy range (-4 to -1.5 eV). The third region above E_F_ level is dominated by minority spin states of Fe-3d occupying the empty levels of the conduction band. Moreover, the Fe-3d and Pt-5d states hybridization for majority spin becomes stronger than that for minority spin. One can see clearly that the 3d states of Fe atoms lie on the higher-energy side of the 5d states of Pt atoms because these states lie slightly on the lower energy side of Fe-3d states as the difference between the Fe and Pt is two electrons per atom.

**Figure 12:**
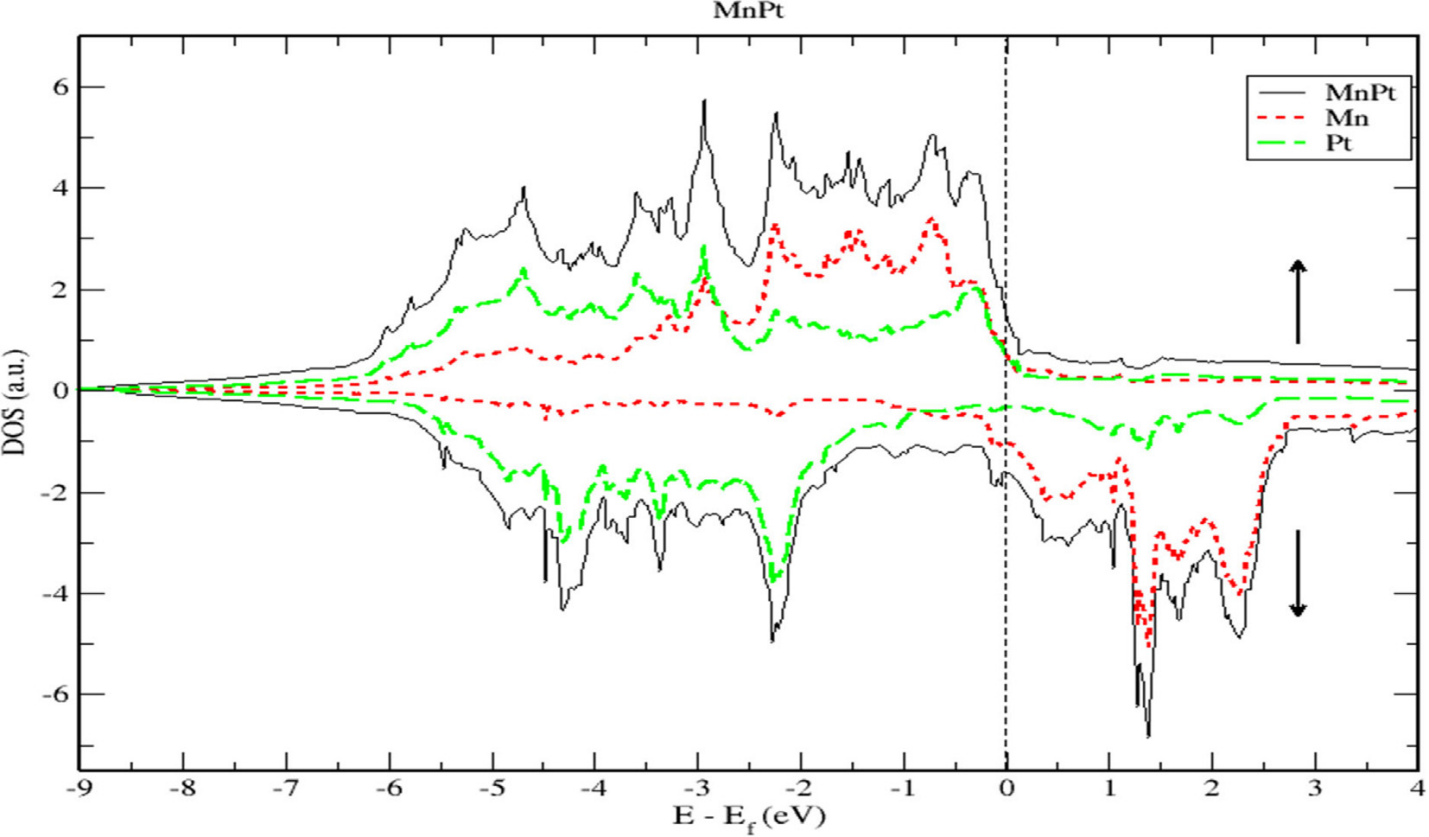
Density of state of MnPt binary. The dashed lines represent partial density of states (PDOS) of MnPt. The vertical line corresponds to the Fermi energy.

**Figure 13:**
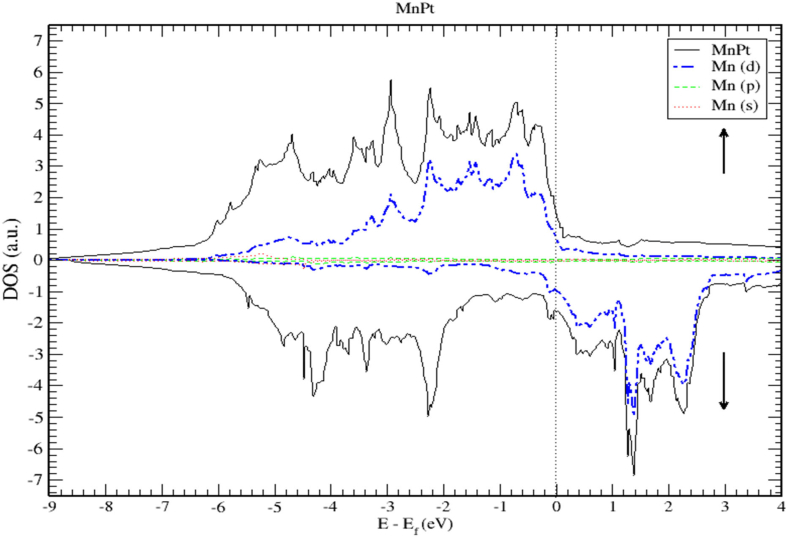
Density of state of MnPt binary. The dashed lines represent partial density of states (PDOS) of Mn. The vertical line corresponds to the Fermi energy.

**Figure 14:**
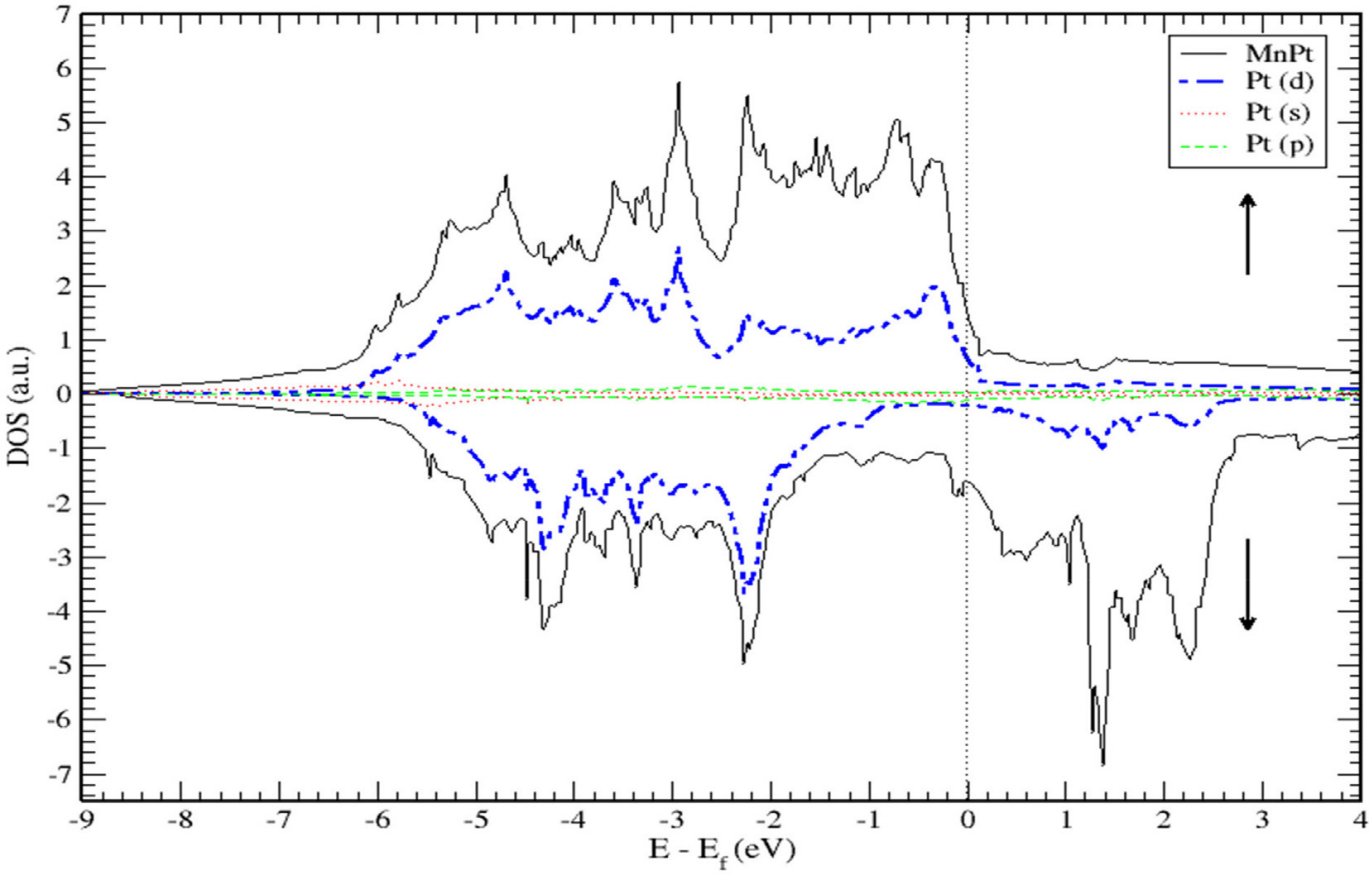
Density of state of MnPt. The dashed lines represent partial density of states (PDOS) of Pt. The vertical line corresponds to the Fermi energy.

The DOS and PDOS plots of MnPt ordered system are shown in Figures 12, 13, and 14. The figures illustrate the majority and minority spin states, respectively. Inspection of Figure 12 demonstrates clearly that the MnPt has an extremely low TDOS to the right of E_F_ level. As seen before for FePt, the contributions to TDOS of MnPt come mainly from Mn-3d and Pt-5d electronic states and it depends largely on the contribution of Mn-3d states. The contributions of s and p electronic states are negligible as illustrated in Figures 13 and 14. The width of the majority band is ~6 eV narrower than the corresponding energy width of the minority band (~8.5 eV) indicating a larger DOS in the majority bands than the corresponding DOS in the minority bands.

The *d* bands of both Mn and Pt significantly overlap below the E_f_ in the majority spin state as shown in Figure 12. This mean that both d bands in the majority spin state are strongly hybridized indicating that the ferromagnetic spin configuration of MnPt originates from the d-d hybridization between d states of Mn and Pt, while in the minority spin state, the d bands of Pt and Mn form bonding and anti-bonding states respectively, this is where hybridization comes into place analogous to what we found for FePt alloy.

**Figure 15:**
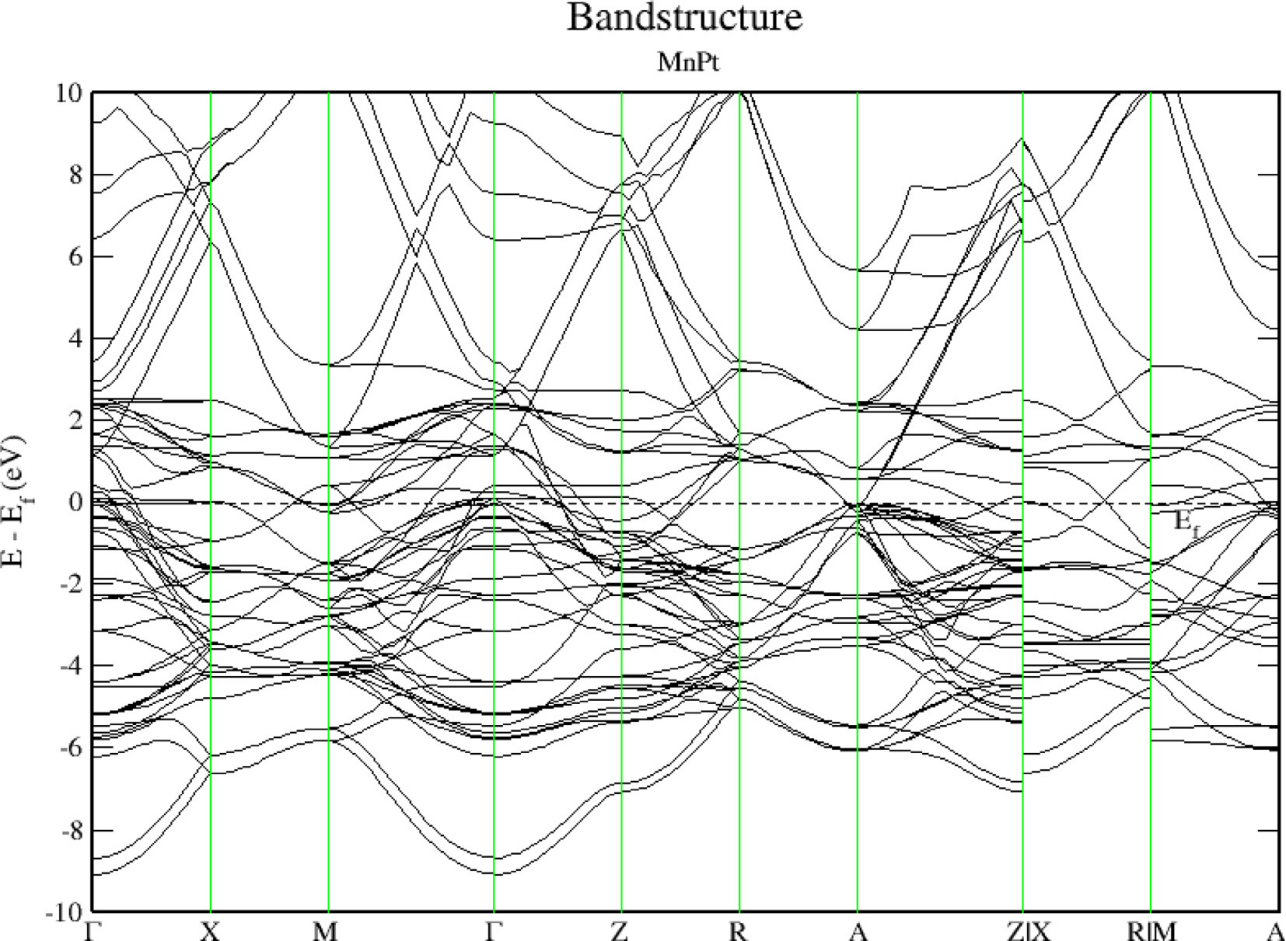
Electronic band structure of the MnPt system. Band gap is missing, and bandgap energy is identically zero.

**Figure 16:**
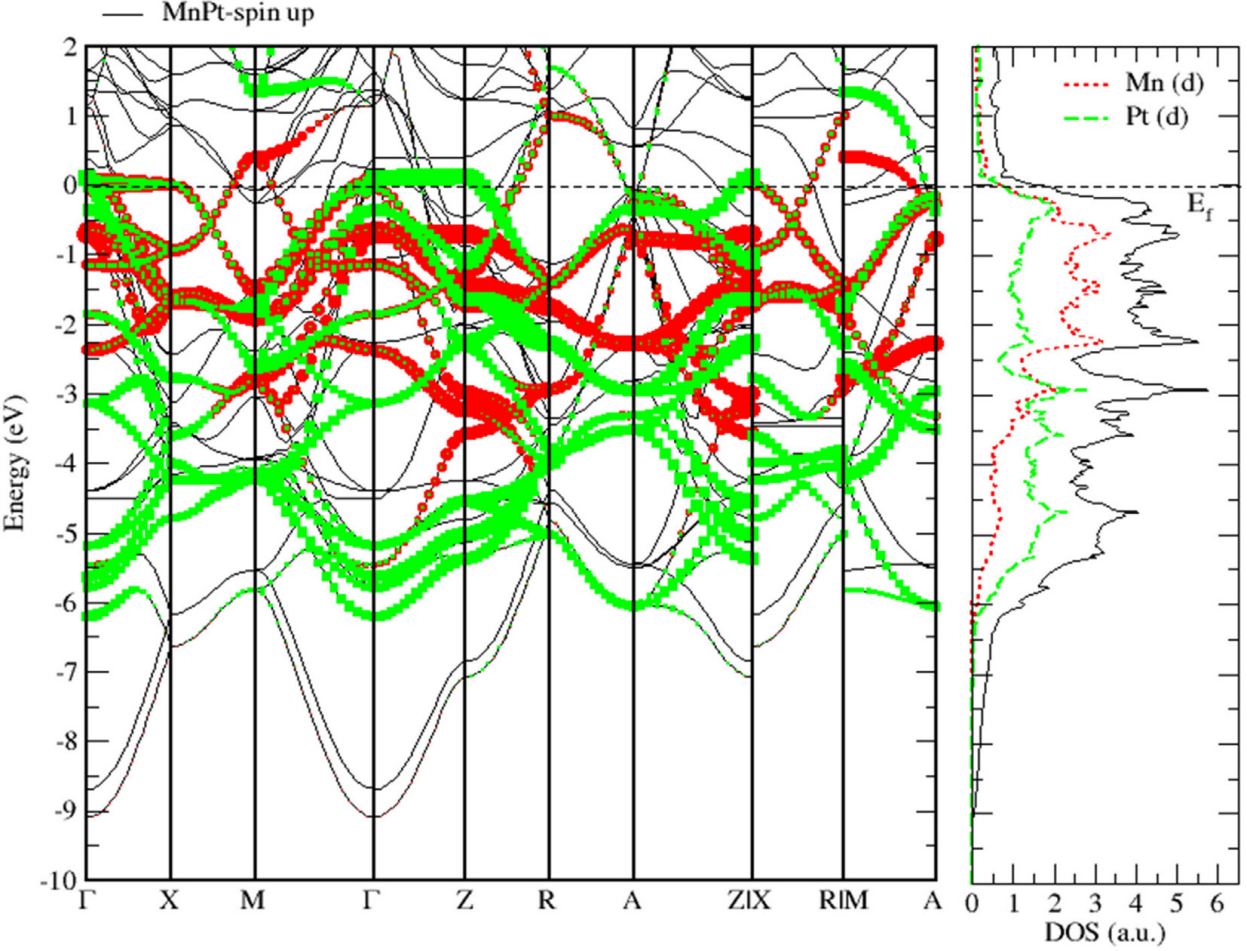
Band structure and spin up PDOS of MnPt binaries.

**Figure 17:**
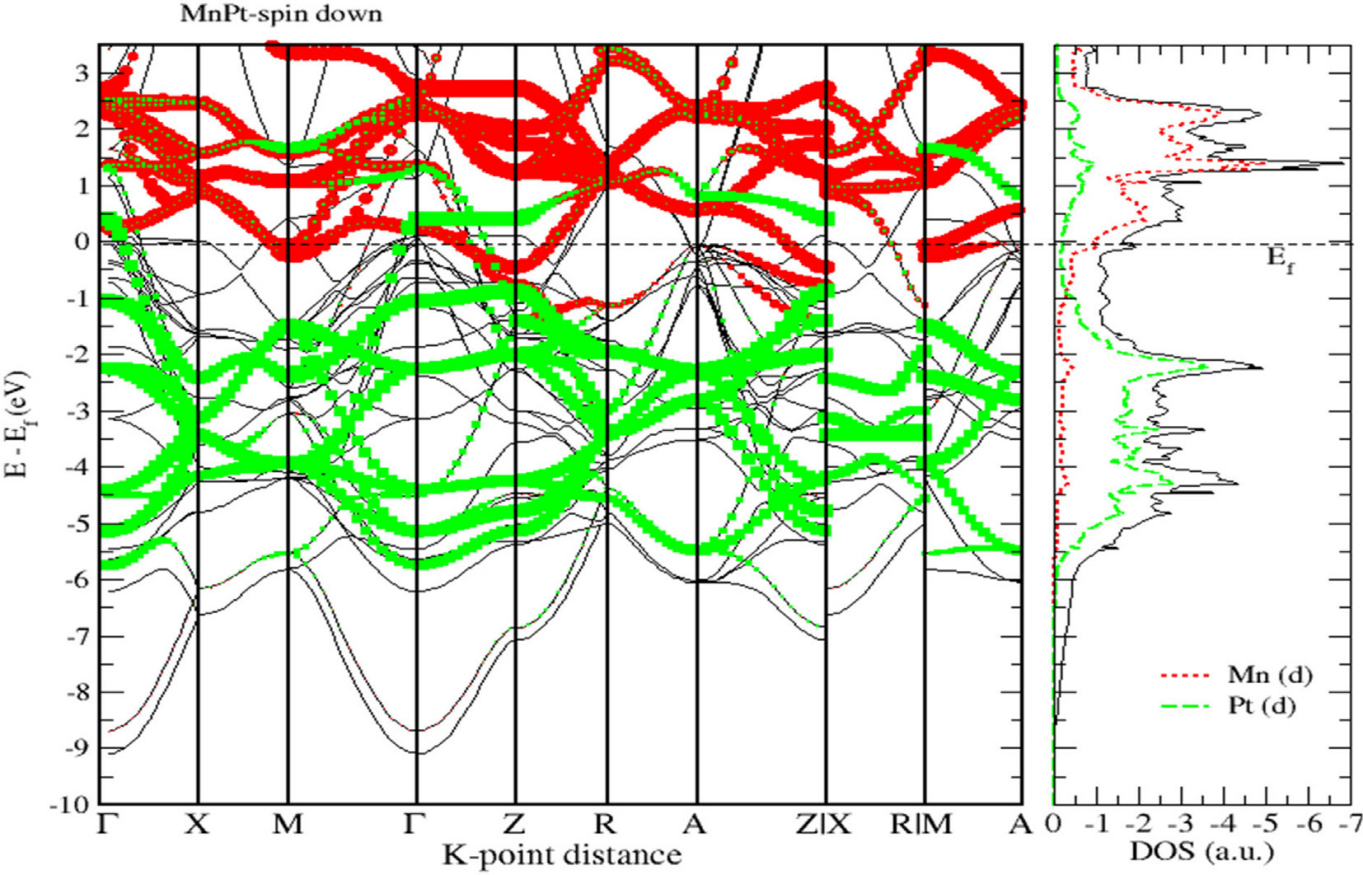
Band structure and spin down PDOS of MnPt binaries.

As seen before for FePt, Figure 15 shows that MnPt system has zero bandgap indicating that it is a pure metallic compound. Figures 16 and 17 indicate that the Mn-3d and Pt-5d states hybridization for majority spin is considerably stronger than the corresponding hybridization for minority spin states.

Careful inspection of Figures 6, 7, 8, 10, 11, 12, 13, 14, and 16 that display L1_0_ FePt and MnPt ordered binaries (TDOS and PDOS). Our results indicate that Fermi level of ordred MnPt alloy is slightly higher than that of ordered FePt alloy. For both ordered FePt and MnPt alloys, a distinctive characteristic of spin up is that it has an extremely deep TDOS minimum. The d states of Pt exhibit lower energy than d states of Fe. This could be interpreted in terms of the difference in number of electrons per atom (2 electrons/atom). The Pt, Mn and Fe atoms exhibit magnetic moments of zero, 4 and ~3, respectively. The d states of Mn and Fe atoms are found to mainly exhibit degenerate local majority states. Furthermore, the AFM of Mn is a result of local minority spin channel of Mn d states that have no adjacent similar state of the same energy for hybridization to take place. The energy interfaces between the Mn-local minority d states the adjacent states of same energy in the same spin channel are positive. Consequently, E_Mn↓_ d states are relatively higher in energy.

**Figure 18:**
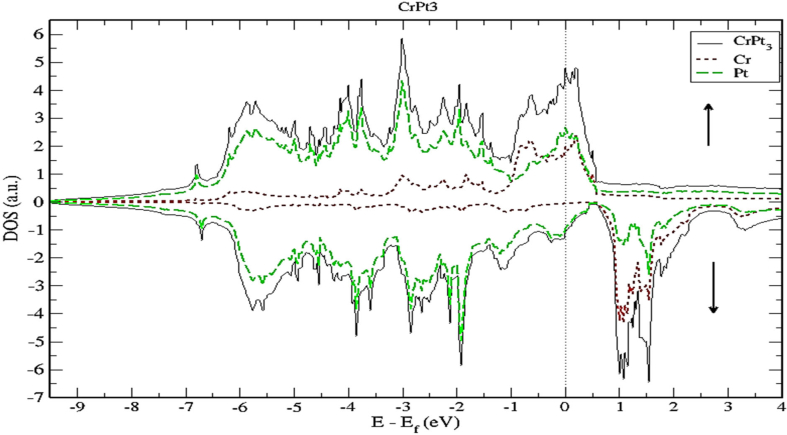
Density of state of CrPt_3_. The dashed lines represent partial density of states (PDOS) of CrPt_3_. The vertical dashed line corresponds to the Fermi energy.

**Figure 19:**
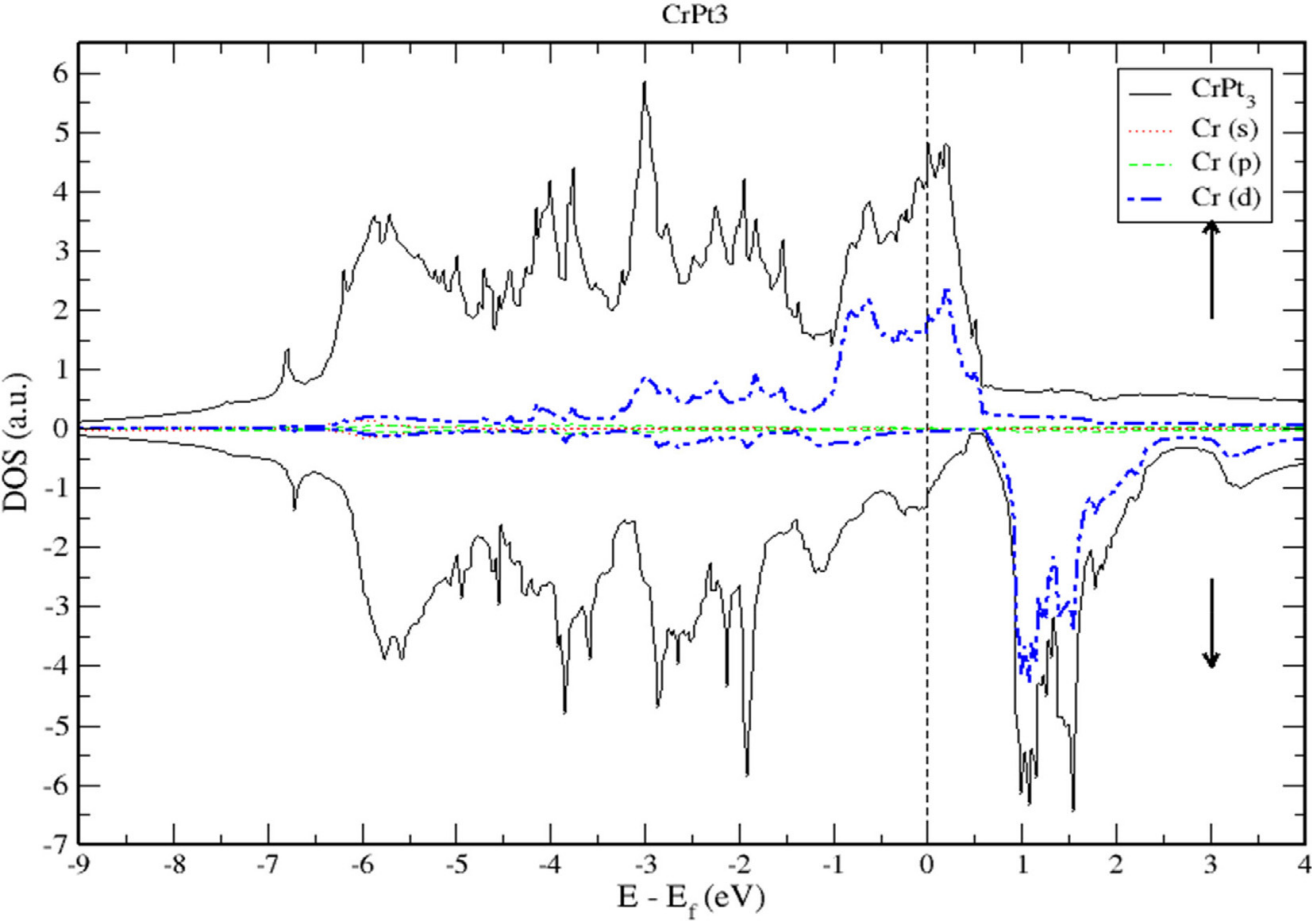
Density of state of CrPt_3_ binary. The dashed lines represent partial density of states (PDOS) of Cr. The vertical line corresponds to the Fermi energy.

As demonstrated by Figures 18 and 19, the contribution of s and p electrons to the density of states of Cr and Pt is negligibly small. Therefore, the electrons of Cr-3d and Pt-5d are mainly responsible for the structural properties of the CrPt_3_ compound. A careful inspection of TDOS of CrPt_3_ reveals a large peak in the DOS of minority spin state that formed from the Cr-3d states above Fermi level. This minority peak originates mainly from Cr-3d bands with much smaller contributions from Pt-5d derived states as illustrated in Figures 17 and 19. The major peaks of this compound come mostly from the electronic states of 5d-Pt atoms. We found that the width of the majority band ~7 eV is narrower than the corresponding energy width of the minority band ~ 9eV indicating that the DOS is larger in the majority bands than in the minority bands. Analogous to the discussion of the band width of DOS of FePt and MnPt, the orbitals are strongly localized and the effective mass is higher leading to a large number of states over a small energy range.

**Figure 20:**
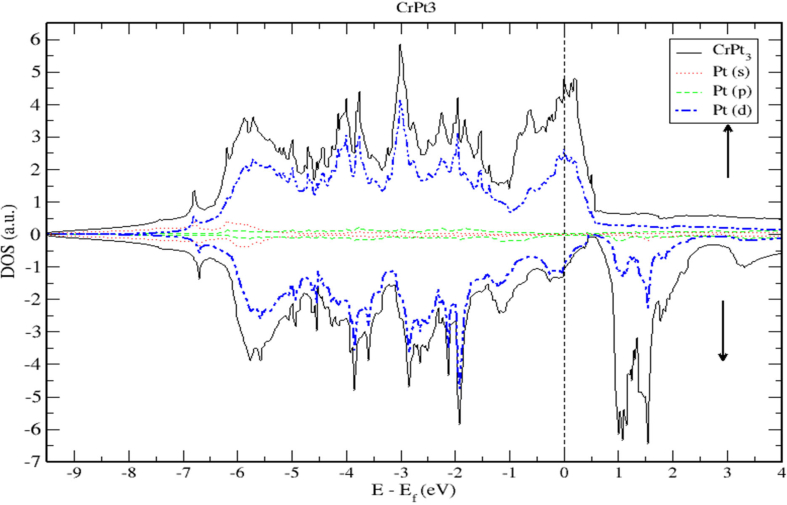
Density of state of CrPt_3_ binary. The dashed lines represent partial density of states (PDOS) of Pt. The vertical line corresponds to the Fermi energy.

As shown in Figure 18, the 3d states of the Cr atoms located near the top of the Pt-5d band form relatively narrow 3d bands. The hybridization of Cr-3d and Pt-5d states can be interpreted quantatively by calculating the PDOS. By inspecting Figure 19 near the Fermi energy, we notice that Pt-d densities follow distinctly the Cr-d densities, a clear sign of strong hybridization (see Figure 20).

**Figure 21:**
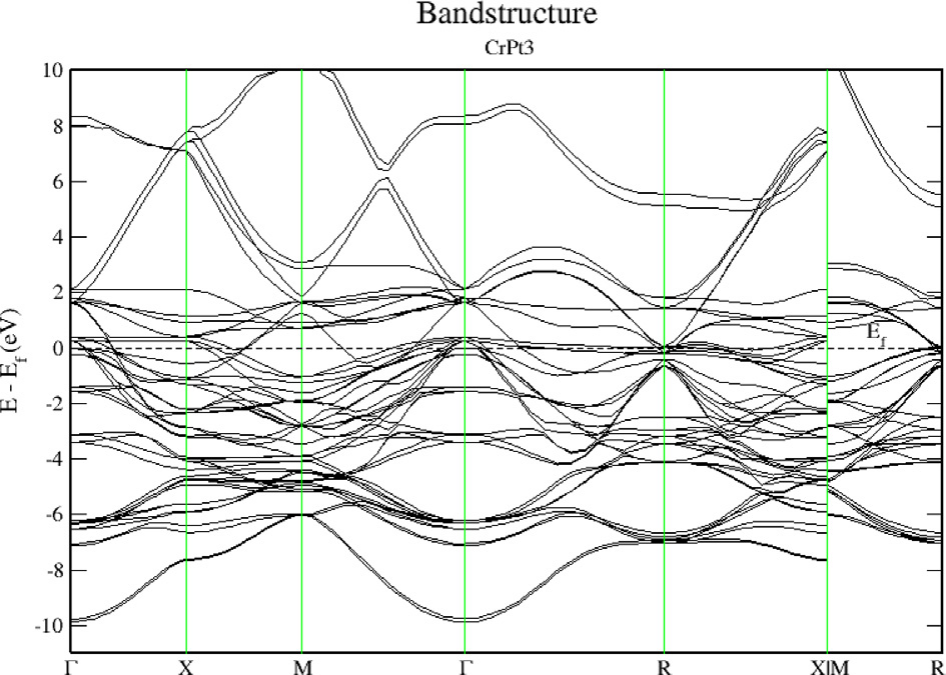
Electronic band structure of the CrPt_3_ ordered alloys. Band gap is missing, and bandgap energy is identically zero.

**Figure 22:**
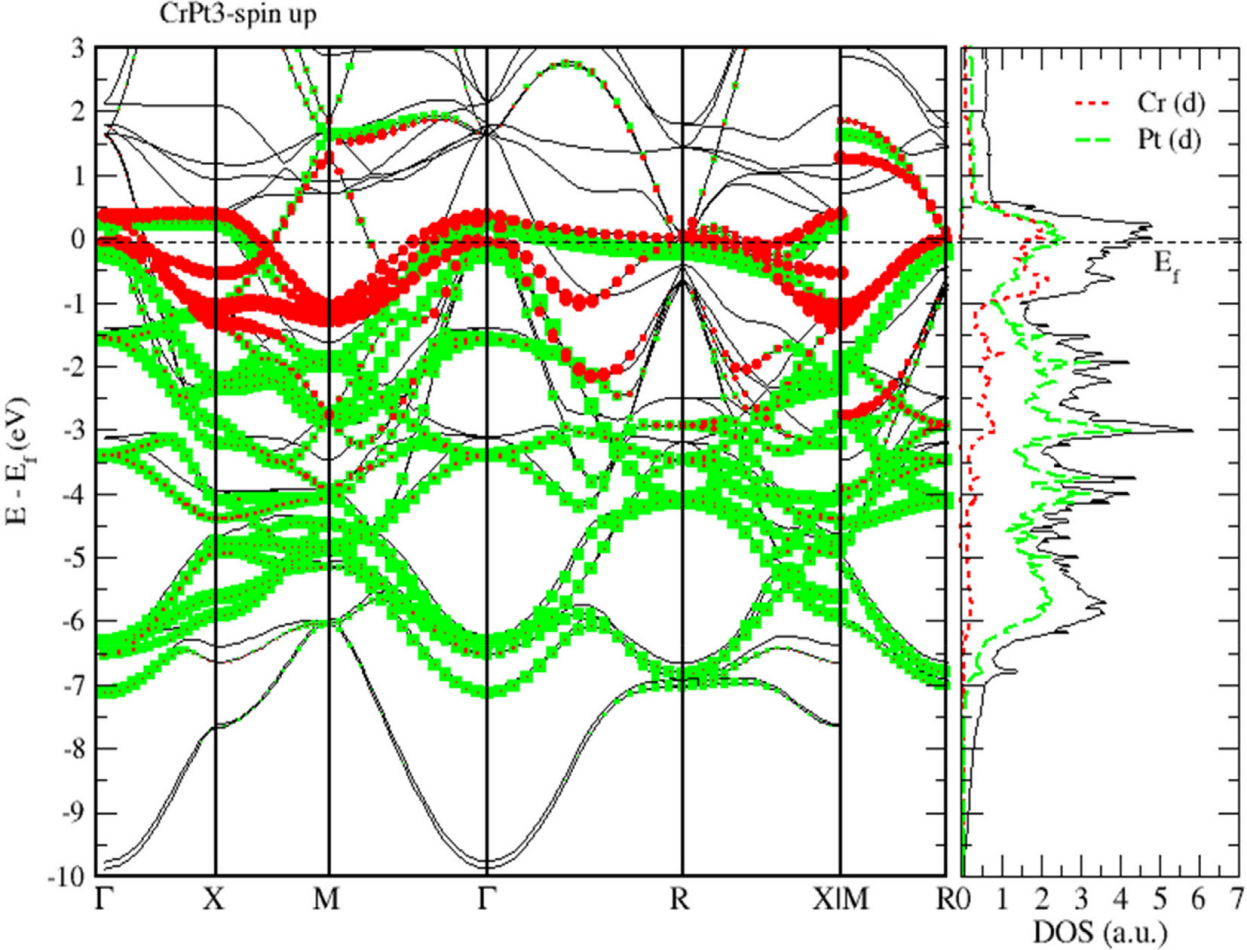
Band structure and spin up PDOS of CrPt_3_ alloys.

**Figure 23:**
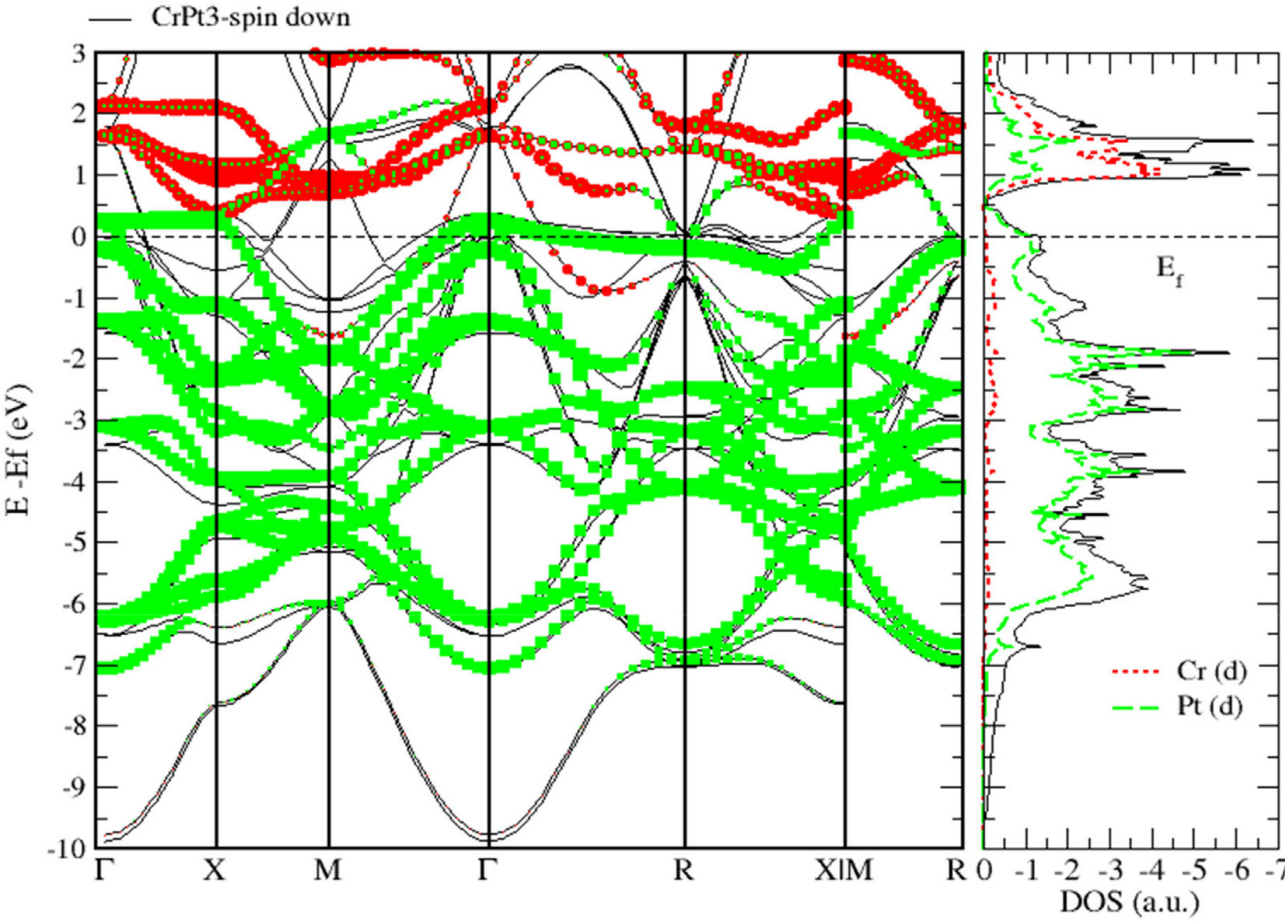
Band structure and spin down PDOS of CrPt_3_ alloys.

The electronic band structure of CrPt_3_ system is displayed in Figure 21 demonstrating that the CrPt_3_ system has zero bandgap. As shown in Figures 22 and 23, the Pt 5d states lie below the 3d states of the Cr atoms on the lower energy side. The Cr-3d bands with spin-up and spin-down shift to the lower- and higher energy sides, respectively. The hybridization between the Cr-3d and Pt-5d states with spin-up becomes stronger than that with down spin. This refers to the number of d holes on Pt in the spin-up bands that are greater than in the spin-down bands.

Having interpreted the TDOS and PDOS of the three ordered systems, we then focus our attention on the interplay between electronic properties and magnetic properties in the following section of the paper.

### 3.3 Magnetocrystalline anisotropy energy

In order to study the MAE of both L1_0_ of FePt and MnPt, we include spin-obit coupling. For both binaries, our calculations predict that, from energy considerations, the magnetic moments of MnPt and FePt ordered alloys oriented preferentially in the 001 plane. Magnetic moment orientation shifts from a perpendicular plane to another parallel plane have been reported for specific composition range of L1_0_ stoichiometry. Such experimental abrupt response of MAE to slight variations of composition above 1:1 equiatomic composition is the main reason of the small MAE value of MnPt ordered binary found to be 0.46 meV/f.u. for MnPt system. Our results indicate that MAE is very sensitive to the details of first principles calculations. For instance, MAE can be greatly altered for small variations of lattice constants and axial ratio in the supercell. We employed accurately calculated lattice parameters for both systems to ensure a high degree of accuracy in calculating MAE values.

In crystals, physical properties are generally anisotropic as they exhibit different values and behaviors along different crystallographic directions. The magnetic moment in a single crystal is principally aligned toward the easy direction. The easy and hard directions arise from the interaction of the spin magnetic moment with the crystal lattice (spin–orbit coupling). Accurate calculation of magnetic anisotropy in crystals is very important as its value is considered to be vital in current and future technological applications of spintronic and magneto-optical devices. In this study, we report MAE values of FePt, MnPt and CrPt_3_ binaries calculated as described in the computational details section. Our results agree well with the previous experimental and theoretical studies within numerical precision.

As indicated by Table 2, the L1_0_ phase of FePt exhibits larger MAE and K values than the corresponding values of L1_0_ MnPt. However, the total magnetic moment of MnPt is larger than that of L1_0_ phase of FePt. Furthermore, we found that CrPt_3_ exhibit an abnormally large MAE and K values for a typical compound adopts a cubic phase. It also exhibits a total magnetic moment of 2.51${\text{μ}}_{B}$. The smaller values of MAE and K in MnPt and CrPt_3_ binaries compared to L1_0_ FePt binary are due to a relatively weaker exchange splitting. In general, total magnetic moment in the investigated binary systems depends on the filling of electrons in 3d orbital of (Fe, Mn, and Cr) atoms [Fe (3d^7^ 4s^1^), Mn (3d^6^ 4s^2^) and Cr (3d^5^ 4s^1^)].

**Table 2:** The computed values of MAE, anisotropy constant K, the total magnetic moment of FePt, MnPt and CrPt_3_ binaries Using LSDA + U and force theorem methods.

Material	MAE (meV/f.u.)	MAE_th_ (meV/f.u.)	MAE_ex_ (meV/f.u.)	Anisotropy constant K (erg/cm^3^)	Total magnetic moment $\left({\text{μ}}_{B}\right)$
L1_0_-FePt	2.66	2.9^a^	2.45–3.16^b^	7.6 × 10^7^	6.34
L1_0_-MnPt	0.46	0.51^c^	0.51^d^	1.3 × 10^7^	7.86
L1_2_-CrPt3	0.42	0.55^e^	0.3^f^	1.1 × 10^7^	2.51

a Ref. [1, 51].

b Ref. [31, 64].

c Ref. [32].

d Ref. [65].

e Ref. [2].

f Ref. [66].

We used LSDA + U method to calculate the spin (Ms), orbital (M_l_) magnetic moments of 3d and 5d states, as well as, total magnetic moment (M_tot_) per formula unit (μB) of FePt, MnPt and CrPt_3_ ordered binary alloys. The obtained results are presented in Table 3. The calculated MAE (meV/f.u.) values of ordered L1_0_ FePt, L1_0_ MnPt and L1_2_ CrPt_3_ are found to be 2.66, 0.46 and 0.42 respectively. Our results of MAE of the three investigated ordered alloys are found to be in good agreement with previous works [2, 32, 48]. The origin of the abnormally large MAE perceived for crystalline CrPt_3_ is due to the large orbital moment of 0.149 μB created on Cr parallel to the spin moment of 2.563 μB. This abnormal property arises from strong Cr 3d–Pt 5d hybridization as can be clearly seen from the DOS and PDOS plots (Figures 22 and 23). This unusually strong hybridization leads to a relatively large computed MAE of 0.48 meV/f.u. that is reasonably larger than the room-temperature experimental value of 0.3 meV/f.u. In addition, we estimated the Curie temperatures of the three ordered alloys from the exchange energy data extracted. The high T_C_ of L1_0_ FePt, MnPt and L1_2_ CrPt_3_ enforces severe requirements on the media and optical system for Heat-Assisted Magnetic Recording [67, 68, 69].

**Table 3:** The calculated spin magnetic moment (M_s_), orbital magnetic moment (M_l_) and total magnetic moment (M_tot_) of the individual atoms of the ordered binary FePt, MnPt, and CrPt_3_ alloys using LSDA + U method for different directions.

Atom	Direction	Spin Moment M_s_ (μ_B_)	Orbital Moment M_l_ (μ_B_)	Total Moment M_tot_ (μ_B_)
Fe	100	2.818	0.078	2.896
	001	2.810	0.078	2.888
Pt	100	0.404	0.068	0.472
	001	0.340	0.056	0.396
Mn	100	3.568	0.038	3.606
	110	3.546	0.029	3.575
Pt	100	0.390	0.034	0.424
	110	0.363	0.026	0.389
Cr	001	2.563	0.149	2.712
	110	2.563	0.149	2.712
Pt	001	-0.031	−0.05	-0.081
	110	-0.031	−0.05	-0.081

From the exchange integral of the short range ordered neighboring atoms, we could estimate Curie temperature (T_C_) of FePt, MnPt, and CrPt_3_ alloys. The exchange integral J and T_C_ can be related to each other using Heisenberg model and the mean field model [70, 71],

$$T_{C}=\frac{2}{3K_{B}}\;s\left(s+1\right)\;\left(z{\;}_{1}\;J_{1}+z_{2}\;J_{2}\right)$$

where k_B_ is the Boltzmann constant, J_1_, J_2_ are the first, second nearest neighbor exchange constants and z_1_, z_2_ are the number of the first and second nearest neighbor atoms, respectively.

As listed in Table 4, the calculated values of T_C_ are in good agreement with previous theoretical values for L1_0_-FePt, L1_0_-MnPt and L1_2_-CrPt_3_ ordered binary alloys. However, we found a considerable discrepancy between the reported theoretical and experimental values of *T*_*C*_. Therefore, it would be very interesting to compare the different theoretical and experimental results of T_C_ of the three investigated ordered alloys presented in Table 4. The differences between theoretical and experimental *T*_*C*_ values are still significant and they may be attributed to thermal spin fluctuations, since there is an experimental evidence of such spin fluctuations. Current development in the storage density of magnetic recording needs media containing extremely small magnetic grains featured by thermally stable magnetization with extremely high anisotropies. The Heat-Assisted Magnetic Recording (HAMR) technology has attracted the attention of researchers investigating optimum thermal conditions for high efficient FePt magnetic grains in scaled magnetic recording. (HAMR) is a swiftly emerging technology, aimed to revolutionize magnetic recording. The high Curie temperatures *Tc* obtained for the three investigated magnetic systems make them appropriate potential candidates to serve as write assist in HAMR magnetic media schemes. Furthermore, we investigate the behavior of magnetization of FePt ordered alloys in the vicinity of *Tc.* Due to the distinct features of L1_0_ FePt phase, such as its low bulk Tc and large anisotropy constant K presently regarded as the best contender for HAMR magnetic media. Actual application of HAMR involves understanding the high-temperature phase transition behavior of FePt, including critical exponents (β) and *Tc* diffusions as the self-motivated HAMR media scheme entails. The L1_0_ FePt phase has been reported to exhibit huge uniaxial anisotropy. This is a critical feature mandatory to boost thermal stability of FePt magnetic grains. Throughout the operation of HAMR unit based on FePt, the high anisotropy of FePt is overwhelmed during magnetic recording by energetic laser that causes the heating of the magnetic grains close to their Tc. A deeper understanding of the dependence of *Tc* on the size of FePt magnetic grains in granulated films is crucial for evolving HAMR technology. β describes quantatively the divergent performance of thermal behavior in L1_0_ FePt near *Tc*. It describes the behavior of physical quantities near Tc. It is used in a diversity of applications of high-temperature magnetism.

**Table 4:** The calculated Curie temperature (T_C_) of the three ordered magnetic binaries in kelvin.

Material	(Tc) K	(Tc)_ex_ K	(Tc)_th_ K
L1_0_-FePt	955	710 [72]	930 [30]
L1_0_-MnPt	989	975 [36]	970 [69]
L1_2_-CrPt_3_	762	494 [25,69]	834 [68]

In the vicinity of *T*_*C*_ and near the Curie temperature, magnetization can be expressed as,

(1) $$M\left(T\right)\alpha {\left(T_{c}-T\right)}^{\beta }.$$

Eq. (1) demonstrates the temperature dependence of magnetization very close to *Tc*, where β stands for the critical exponent that depends on the type of the magnetic interaction. We consider the finite-size scaling analysis adopted in [73, 74, 75] to calculate βof L1_0_ FePt phase. To investigate the phase transition behavior of FePt, we consider the realistic effective spin Hamiltonian,

(2) $$H=\;-\sum_{ij}\left(J_{ij}\;S_{i}\;\cdot S_{j}+d_{ij}^{\left(2\right)}S_{i}^{Z}\;S_{j}^{Z}\right)-\sum_{i}d_{i}^{\left(0\right)}{\left(S_{i}^{Z}\right)}^{2}+\mu_{Fe}HS_{i}^{Z}$$

Eq. (2) accounts for all the possible interactions in high uniaxial anisotropic L1_0_ FePt phase. In this equation, $\mu_{Fe}$ = 3.23 $\mu_{B}$ is the magnetic moment of Fe atom. $\mu_{B}$ = 9.274 × 10^−24^J/T is the Bohr magneton and H stands for the strength of the magnetic field directed along the easy z-axis. The effective exchange interaction $J_{ij}$ includes all possible interactions up to the second nearest-neighbors. To simulate the magnetic field and temperature dependent magnetization data of the model defined by Eq. (2), we use SLDA described in methodology section. We treated Fe magnetic grains as spherical particles with radii *R* in the range of 1–8 nm. The magnetic field strengths are specifically selected in the range 0–3 meV (0–16 T) and the temperature T ranges from 300 to 1000 K. We calculated β to be 0.365 consistent with the value reported by [76, 77] within numerical accuracy.

## 4 Conclusions

In summary, First-principles structural stability investigations show that both FePt and MnPt exhibit the face centered tetragonal L1_0_ (CuAu) structure labeled as L1_0_ phase, whereas the most stable phase of CrPt_3_ is the cubic L1_2_ (CuAu_3_). The interplay among electronic band structure, total spin density of states (TDOS), spin partial density of states (PDOS) and magnetic properties is examined and interpreted. We found that L1_0_ FePt, L1_0_ MnPt and L1_2_ CrPt_3_ exhibit MAE (K) values of 2.66 meV/f.u (1.7 × 10^7^ erg/cm^3^), 0.46 meV/f.u (1.3 × 10^7^ erg/cm^3^) and 0.42 meV/f.u (1.1 × 10^7^ erg/cm^3^), respectively. To clarify the influence of each atom to electronic and magnetic properties, we calculated the (TDOS) and (PDOS) of each alloy. We found that the major contribution to the (TDOS) comes from hybridization of M-3d and Pt-5d states (M = Fe, Mn). The preponderance of this kind of hybridization is predicted to boost both spin and orbital magnetic moments and thus enhances the magnetic properties. In L1_0_ FePt, this hybridization is strong enough to enhance the MAE and K values significantly. The smaller values of MAE and K in L1_0_ MnPt are attributed to the weak hybridization between Mn-3d and Pt-5d states. The small values of MAE and K of L1_2_ CrPt_3_ are attributed to the weaker hybridization between Cr-3d and Pt-5d states. Furthermore, we estimated the Curie temperature T_c_ of the three alloys from the exchange energy data. The T_c_ values of L1_0_ FePt, L1_0_ MnPt and L1_2_ CrPt_3_ are found to be 955 K, 989 K and 762 K, respectively. The noticeable discrepancy between the calculated and experimental Curie temperatures could be attributed partially to the overestimation of the inter-sites interactions and to some extent to thermal spin fluctuations. We examined the behavior of magnetization of FePt in the vicinity of T_C_, we found the critical exponent of L1_0_ FePt to be about 0.37 that makes this phase the most appropriate for HAMR. The outstanding electronic and magnetic properties of L1_0_ FePt, L1_2_ CrPt_3_ and L1_0_ MnPt ordered alloys indicate that they could be used in the fabrication of magneto-optical devices with ultrahigh magnetic storage, as well as, for perpendicular magnetic data recording and magnetic actuators.

## Declarations

### Author contribution statement

A. M. Alsaad: Conceived and designed the experiments; Performed the experiments; Analyzed and interpreted the data; Contributed reagents, materials, analysis tools or data; Wrote the paper.

A. A. Ahmad: Conceived and designed the experiments; Analyzed and interpreted the data; Contributed reagents, materials, analysis tools or data; Wrote the paper.

Tareq S. Obeidat: Conceived and designed the experiments; Performed the experiments; Contributed reagents, materials, analysis tools or data.

### Funding statement

This work was supported by Jordan University of Science and Technology.

### Competing interest statement

The authors declare no conflict of interest.

### Additional information

No additional information is available for this paper.

## References

[1] MacLaren J.M., Duplessis R.R., Stern R.A., Willoughby S. (2005). First principles calculations of FePt, CoPt, Co/sub 3/Pt, and Fe/sub 3/Pt alloys. IEEE Trans. Magn..

[2] Oppeneer P.M., Galanakis I., Grechnev A., Eriksson O. (2002). Unusual magnetism and magnetocrystalline anisotropy of CrPt3. J. Magn. Magn Mater..

[3] Krishnan K.M., Nelson C., Echer C., Farrow R., Marks R., Kellock A. (1998). Exchange biasing of permalloy films by Mn x Pt 1− x: role of composition and microstructure. J. Appl. Phys..

[4] Futamoto M., Nakamura M., Ohtake M., Inaba N., Shimotsu T. (2016). Growth of L 10-ordered crystal in FePt and FePd thin films on MgO (001) substrate. AIP Adv..

[5] Ujihara M., Lee D.G., Carman G.P. (2010). Energy harvesting by means of thermo-mechanical device utilizing bistable ferromagnets. Google Patents.

[6] Tsang S.C., Yu C.H., Tang H., He H., Castelletto V., Hamley I.W., Narayanan T., Lo C.C., Tam K. (2008). Assembly of centimeter long silica coated FePt colloid crystals with tailored interstices by magnetic crystallization. Chem. Mater..

[7] Hao R., Xing R., Xu Z., Hou Y., Gao S., Sun S. (2010). Synthesis, functionalization, and biomedical applications of multifunctional magnetic nanoparticles. Adv. Mater..

[8] Guhr I., Riedlinger B., Maret M., Mazur U., Barth A., Treubel F., Albrecht M., Schatz G. (2005). Structural and magnetic properties of CrPt 3 (111) films grown on WSe 2 (0001). J. Appl. Phys..

[9] Gubin S.P. (2009). Magnetic Nanoparticles.

[10] Hanyu T., Endoh T., Ando Y., Ikeda S., Fukami S., Sato H., Koike H., Ma Y., Suzuki D., Ohno H. (2019). Spin-transfer-torque magnetoresistive random-access memory (STT-MRAM) technology. Advances in Non-volatile Memory and Storage Technology.

[11] Ando K., Fujita S., Ito J., Yuasa S., Suzuki Y., Nakatani Y., Miyazaki T., Yoda H. (2014). Spin-transfer torque magnetoresistive random-access memory technologies for normally off computing. J. Appl. Phys..

[12] Hao L., Granata C. (2017). Recent trends and perspectives of nanoSQUIDs: introduction to ‘Focus on nanoSQUIDs and their applications’. Supercond. Sci. Technol..

[13] Dwivedi N., Ott A., Sasikumar K., Dou C., Yeo R., Narayanan B., Sassi U., De Fazio D., Soavi G., Dutta T. (2019). Graphene overcoats for ultra-high storage density magnetic media. arXiv preprint arXiv:1906.00338.

[14] Xiaoyu L., Sharma P., Zhang Y., Makino A., Kato H. (2019). Nano-imprinting potential of magnetic FeCo-based metallic glass. Nanotechnology.

[15] Miyazawa K., Okamoto S., Yomogita T., Kikuchi N., Kitakami O., Toyoki K., Billington D., Kotani Y., Nakamura T., Sasaki T. (2019). First-order reversal curve analysis of a Nd-Fe-B sintered magnet with soft X-ray magnetic circular dichroism microscopy. Acta Mater..

[16] Sales B.C., Saparov B., McGuire M.A., Singh D.J., Parker D.S. (2014). Ferromagnetism of Fe 3 Sn and alloys. Sci. Rep..

[17] Dai D., Xiang H., Whangbo M.H. (2008). Effects of spin-orbit coupling on magnetic properties of discrete and extended magnetic systems. J. Comput. Chem..

[18] Kota Y., Sakuma A. (2012). Relationship between magnetocrystalline anisotropy and orbital magnetic moment in L 10-type ordered and disordered alloys. J. Phys. Soc. Jpn..

[19] Sakuma A. (1994). First principle calculation of the magnetocrystalline anisotropy energy of FePt and CoPt ordered alloys. J. Phys. Soc. Jpn..

[20] Bruno P. (1989). Tight-binding approach to the orbital magnetic moment and magnetocrystalline anisotropy of transition-metal monolayers. Phys. Rev. B.

[21] Jansen H. (1988). Magnetic anisotropy in density-functional theory. Phys. Rev. B.

[22] Galanakis I., Alouani M., Dreyssé H. (2000). Perpendicular magnetic anisotropy of binary alloys: a total-energy calculation. Phys. Rev. B.

[23] Li D. (2015). Magneto-crystalline anisotropy of metallic nanostructures: tight-binding and first-principles studies. Université Pierre et Marie Curie-Paris VI.

[24] Lu Z., Chepulskii R.V., Butler W. (2010). First-principles study of magnetic properties of L 1 0-ordered MnPt and FePt alloys. Phys. Rev. B.

[25] Patel R., Liddiard A., Crapper M. (1994). Studies of the electronic structure of the ferrimagnetic alloy Pt3Cr using normal take-off angle-resolved photoemission. J. Phys. Condens. Matter.

[26] Severin C., Chen C., Stassis C. (1979). Neutron-diffraction study of the magnetic structure of MnPt alloys. J. Appl. Phys..

[27] Lyubina J., Opahle I., Richter M., Gutfleisch O., Müller K.-H., Schultz L., Isnard O. (2006). Influence of composition and order on the magnetism of Fe–Pt alloys: neutron powder diffraction and theory. Appl. Phys. Lett..

[28] Aas C., Szunyogh L., Chantrell R. (2013). Effects of composition and chemical disorder on the magnetocrystalline anisotropy of FexPt1− x alloys. EPL (Europhysics Letters).

[29] Burkert T., Eriksson O., Simak S.I., Ruban A.V., Sanyal B., Nordström L., Wills J.M. (2005). Magnetic anisotropy of L 1 0 FePt and Fe 1− x Mn x Pt. Phys. Rev. B.

[30] Staunton J., Ostanin S., Razee S., Gyorffy B., Szunyogh L., Ginatempo B., Bruno E. (2004). Long-range chemical order effects upon the magnetic anisotropy of FePt alloys from an ab initio electronic structure theory. J. Phys. Condens. Matter.

[31] Barmak K., Kim J., Lewis L., Coffey K., Toney M., Kellock A., Thiele J.-U. (2005). On the relationship of magnetocrystalline anisotropy and stoichiometry in epitaxial L 1 0 CoPt (001) and FePt (001) thin films. J. Appl. Phys..

[32] Umetsu R., Sakuma A., Fukamichi K. (2006). Magnetic anisotropy energy of antiferromagnetic L 1 0-type equiatomic Mn alloys. Appl. Phys. Lett..

[33] Kato T., Iwata S., Yamauchi Y., Tsunashima S. (2009). Modification of magnetic properties and structure of Kr+ ion-irradiated CrPt 3 films for planar bit patterned media. J. Appl. Phys..

[34] Leonhardt T., Chen Y., Rao M., Laughlin D., Lambeth D., Kryder M. (1999). CrPt 3 thin film media for perpendicular or magneto-optical recording. J. Appl. Phys..

[35] Besnus M., Meyer A. (1973). Magnetic properties of the ordered and disordered CrPt3 and CrPt phases. Phys. Status Solidi.

[36] Krén E., Kádár G., Pál L., Sólyom J., Szabó P., Tarnóczi T. (1968). Magnetic structures and exchange interactions in the Mn-Pt system. Phys. Rev..

[37] Kresse G., Furthmüller J. (1996). Efficiency of ab-initio total energy calculations for metals and semiconductors using a plane-wave basis set. Comput. Mater. Sci..

[38] Kresse G., Hafner J. (1993). Ab initio molecular dynamics for liquid metals. Phys. Rev. B.

[39] Kresse G., Hafner J. (1994). Ab initio molecular-dynamics simulation of the liquid-metal–amorphous-semiconductor transition in germanium. Phys. Rev. B.

[40] Kresse G., Furthmüller J. (1996). Efficient iterative schemes for ab initio total-energy calculations using a plane-wave basis set. Phys. Rev. B.

[41] Kresse G., Joubert D. (1999). From ultrasoft pseudopotentials to the projector augmented-wave method. Phys. Rev. B.

[42] Blöchl P.E. (1994). Projector augmented-wave method. Phys. Rev. B.

[43] Vanderbilt D. (1990). Soft self-consistent pseudopotentials in a generalized eigenvalue formalism. Phys. Rev. B.

[44] Perdew J.P., Zunger A. (1981). Self-interaction correction to density-functional approximations for many-electron systems. Phys. Rev. B.

[45] Perdew J.P., Perdew J.P., Wang Y. (1992). Accurate and simple analytic representation of the electron-gas correlation energy. Phys. Rev. B.

[46] Perdew J.P., Burke K., Ernzerhof M. (1996). Generalized gradient approximation made simple. Phys. Rev. Lett..

[47] Perdew J.P., Burke K., Ernzerhof M. (1996). Generalized gradient approximation made simple. Phys. Rev. Lett..

[48] Khan S.A., Blaha P., Ebert H., Minár J., Šipr O. (2016). Magnetocrystalline anisotropy of FePt: a detailed view. Phys. Rev. B.

[49] Błoński P., Hafner J. (2009). Density-functional theory of the magnetic anisotropy of nanostructures: an assessment of different approximations. J. Phys. Condens. Matter.

[50] Victora R., MacLaren J. (1993). Theory of magnetic interface anisotropy. Phys. Rev. B.

[51] Shick A.B., Mryasov O.N. (2003). Coulomb correlations and magnetic anisotropy in ordered L 1 0 CoPt and FePt alloys. Phys. Rev. B.

[52] Yang I., Savrasov S.Y., Kotliar G. (2001). Importance of correlation effects on magnetic anisotropy in Fe and Ni. Phys. Rev. Lett..

[53] Shick A., Pickett W. (2001). Magnetism, spin-orbit coupling, and superconducting pairing in UGe 2. Phys. Rev. Lett..

[54] Shick A., Liechtenstein A., Pickett W. (1999). Implementation of the LDA+ U method using the full-potential linearized augmented plane-wave basis. Phys. Rev. B.

[55] Press W.H., Teukolsky S.A., Vetterling W.T., Flannery B.P. (2007). Numerical Recipes.

[56] Møller M.F. (1993). A scaled conjugate gradient algorithm for fast supervised learning. Neural Network..

[57] Bylander D., Kleinman L., Lee S. (1990). Self-consistent calculations of the energy bands and bonding properties of B 12 C 3. Phys. Rev. B.

[58] Evarestov R., Smirnov V. (2004). Modification of the Monkhorst-Pack special points meshes in the Brillouin zone for density functional theory and Hartree-Fock calculations. Phys. Rev. B.

[59] Monkhorst H.J., Pack J.D. (1976). Special points for Brillouin-zone integrations. Phys. Rev. B.

[60] Methfessel M., Paxton A. (1989). High-precision sampling for Brillouin-zone integration in metals. Phys. Rev. B.

[61] Paxton A., Methfessel M., Polatoglou H. (1990). Structural energy-volume relations in first-row transition metals. Phys. Rev. B.

[62] Lukashev P.V., Horrell N., Sabirianov R.F. (2012). Tailoring magnetocrystalline anisotropy of FePt by external strain. J. Appl. Phys..

[63] Preußner J., Prins S., Völkl R., Liu Z.-K., Glatzel U. (2009). Determination of phases in the system chromium–platinum (Cr–Pt) and thermodynamic calculations. Mater. Sci. Eng., A.

[64] Liu J., Luo C., Liu Y., Sellmyer D. (1998). High energy products in rapidly annealed nanoscale Fe/Pt multilayers. Appl. Phys. Lett..

[65] Umetsu R.Y., Fukamichi K., Sakuma A. (2006). Electrical and magnetic properties, and electronic structures of pseudo-gap-type antiferromagnetic L10-type MnPt Alloys. Mater. Trans..

[66] Kortright J., Awschalom D., Stöhr J., Bader S., Idzerda Y., Parkin S., Schuller I.K., Siegmann H.-C. (1999). Research frontiers in magnetic materials at soft X-ray synchrotron radiation facilities. J. Magn. Magn Mater..

[67] Kryder M.H., Gage E.C., McDaniel T.W., Challener W.A., Rottmayer R.E., Ju G., Hsia Y.-T., Erden M.F. (2008). Heat assisted magnetic recording. Proc. IEEE.

[68] Galanakis I., Şaşıoğlu E. (2012). Ab-initio calculation of effective exchange interactions, spin waves, and Curie temperature in L2 1-and L1 2-type local moment ferromagnets. J. Mater. Sci..

[69] Wang J., Gao A., Chen W., Zhang X.-D., Zhou B., Jiang Z. (2013). The structural, elastic, phonon, thermal and electronic properties of MnX (X= Ni, Pd and Pt) alloys: first-principles calculations. J. Magn. Magn Mater..

[70] Sato K., Katayama-Yoshida H., Dederichs P. (2003). Curie temperatures of III–V diluted magnetic semiconductors calculated from first-principles in mean field approximation. J. Supercond..

[71] Sy H., Ow M. (1992). Curie temperature for a finite alternating magnetic superlattice. J. Phys. Condens. Matter.

[72] Okamoto H., Massalski T. (1993). Guidelines for binary phase diagram assessment. J. Phase Equil..

[73] Mryasov O.N., Nowak U., Guslienko K.Y., Chantrell R.W. (2005). Temperature-dependent magnetic properties of FePt: effective spin Hamiltonian model. EPL (Europhysics Letters).

[74] Yeomans J.M. (1992). Statistical Mechanics of Phase Transitions.

[75] Goldenfeld N. (1992).

[76] Hovorka O., Devos S., Coopman Q., Fan W., Aas C., Evans R., Chen X., Ju G., Chantrell R. (2012). The Curie temperature distribution of FePt granular magnetic recording media. Appl. Phys. Lett..

[77] Waters J., Kramer D., Sluckin T.J., Hovorka O. (2019). Resolving anomalies in the critical exponents of Fe Pt using finite-size scaling in magnetic fields. Phys. Rev. Appl..

